# OTULIN deficiency in ORAS causes cell type‐specific LUBAC degradation, dysregulated TNF signalling and cell death

**DOI:** 10.15252/emmm.201809324

**Published:** 2019-02-25

**Authors:** Rune Busk Damgaard, Paul R Elliott, Kirby N Swatek, Eamonn R Maher, Polina Stepensky, Orly Elpeleg, David Komander, Yackov Berkun

**Affiliations:** ^1^ Medical Research Council Laboratory of Molecular Biology Cambridge UK; ^2^ Department of Medical Genetics University of Cambridge and Cambridge NIHR Biomedical Research Centre Cambridge UK; ^3^ Hebrew University Hadassah Medical School Jerusalem Israel; ^4^ Department of Bone Marrow Transplantation and Cancer Immunotherapy Hadassah Hebrew University Medical Center Jerusalem Israel; ^5^ Monique and Jacques Roboh Department of Genetic Research Hadassah Hebrew University Medical Center Jerusalem Israel; ^6^ Ubiquitin Signalling Division The Walter and Eliza Hall Institute of Medical Research Parkville Melbourne VIC Australia; ^7^ Department of Medical Biology The University of Melbourne Melbourne VIC Australia; ^8^ Department of Pediatrics Hadassah Hebrew University Medical Center Jerusalem Israel; ^9^ Present address: Department of Biochemistry University of Oxford Oxford UK; ^10^ Present address: Department of Molecular Machines and Signaling Max Planck Institute of Biochemistry Martinsried Germany

**Keywords:** cell death, deubiquitinases, inflammatory disease, TNF signalling, ubiquitin, Genetics, Gene Therapy & Genetic Disease, Immunology

## Abstract

The deubiquitinase OTULIN removes methionine‐1 (M1)‐linked polyubiquitin signals conjugated by the linear ubiquitin chain assembly complex (LUBAC) and is critical for preventing TNF‐driven inflammation in OTULIN‐related autoinflammatory syndrome (ORAS). Five ORAS patients have been reported, but how dysregulated M1‐linked polyubiquitin signalling causes their symptoms is unclear. Here, we report a new case of ORAS in which an OTULIN‐Gly281Arg mutation leads to reduced activity and stability *in vitro* and in cells. In contrast to OTULIN‐deficient monocytes, in which TNF signalling and NF‐κB activation are increased, loss of OTULIN in patient‐derived fibroblasts leads to a reduction in LUBAC levels and an impaired response to TNF. Interestingly, both patient‐derived fibroblasts and OTULIN‐deficient monocytes are sensitised to certain types of TNF‐induced death, and apoptotic cells are evident in ORAS patient skin lesions. Remarkably, haematopoietic stem cell transplantation leads to complete resolution of inflammatory symptoms, including fevers, panniculitis and diarrhoea. Therefore, haematopoietic cells are necessary for clinical manifestation of ORAS. Together, our data suggest that ORAS pathogenesis involves hyper‐inflammatory immune cells and TNF‐induced death of both leukocytes and non‐haematopoietic cells.

## Introduction

Timely activation and resolution of the innate immune response is essential for tissue homeostasis and host defence, and dysregulation of this response may cause autoinflammation (Nathan & Ding, 2010). Autoinflammatory diseases are a heterogeneous group of disorders characterised by spontaneous development of episodic or chronic inflammation in the absence of inflammatory provocation. Causative mutations have been identified in more than 25 genes, most of which regulate cytokine signalling, e.g. interleukin‐1 (IL‐1), type I interferon, or tumour necrosis factor (TNF) signalling, or activation of nuclear factor‐κB (NF‐κB) transcription factors (Manthiram *et al*, 2017).

Key regulatory mechanisms in innate immune signalling rely on protein ubiquitination, which is a versatile post‐translational modification that regulates virtually every aspect of cellular homeostasis (Komander & Rape, 2012). Ubiquitin (Ub) mediates most of its cellular functions through structurally and functionally distinct polyUb signals (Komander & Rape, 2012; Swatek & Komander, 2016). These polyUb chains can be linked via one of the seven Lys (K) residues in Ub (e.g. K48‐linked chains) or via Ub Met1 (M1), forming M1‐linked (also known as linear) chains (Swatek & Komander, 2016).

Ubiquitin chains regulate activation of NF‐κB transcription factors to control inflammation and immunity (Bonizzi & Karin, 2004; Jiang & Chen, 2012; Etzioni *et al*, 2017). K48‐linked chains mediate timed degradation of the inhibitor of κB (IκB) proteins while non‐degradative chains, including K63‐ and M1‐linked chains, serve as recruitment and activation platforms in signalling complexes formed after engagement of pattern recognition receptors (PRRs) and cytokine receptors, e.g. TNF receptor‐1 (TNF‐R1). Receptor stimulation, for example of TNF‐R1, triggers assembly of multi‐protein receptor signalling complexes (RSCs) that include a host of E3 Ub ligases, including TNF receptor‐associated factors (TRAFs), inhibitor of apoptosis proteins (IAPs) and the linear Ub chain assembly complex (LUBAC). These generate K63‐ and M1‐linked Ub chains to facilitate the recruitment of the TGFβ‐activated kinase 1 (TAK1) and IκB kinase (IKK) complexes, respectively (Jiang & Chen, 2012). K63 and M1 Ub linkages can occur in the same Ub polymers (Emmerich *et al*, 2013), facilitating TAK1 and IKK co‐localisation and cross‐activation (reviewed in Hrdinka & Gyrd‐Hansen 2017).

M1‐linked Ub chains are conjugated by LUBAC, which consists of the catalytic subunit HOIP and the adaptors and co‐activators HOIL‐1 and SHARPIN (Kirisako *et al*, 2006; Gerlach *et al*, 2011; Ikeda *et al*, 2011; Tokunaga *et al*, 2011). LUBAC exclusively generates M1‐linked Ub chains (Kirisako *et al*, 2006; Stieglitz *et al*, 2013) and is recruited in a Ub‐dependent manner to many receptors of the immune system, including TNF‐R1, IL‐1 receptor (IL‐1R), CD40, TLRs and NOD2, where it ubiquitinates a host of substrates, including RIPK1, RIPK2, MyD88, IRAKs and NEMO directly or on pre‐existing chains (Hrdinka & Gyrd‐Hansen, 2017).

Functionally, formation of RSCs and activation of the Ub‐ and kinase‐dependent signalling cascades leads to activation of NF‐κB transcription factors and pro‐inflammatory transcriptional programmes (Jiang & Chen, 2012). However, for TNF‐R1, it can also lead to cell death via formation of a TRADD‐, FADD‐, RIPK1/3‐ and caspase‐8‐containing death‐inducing complex, complex‐II (Fuchs & Steller, 2015; Kupka *et al*, 2016b). Recent research has shown that M1‐linked Ub chains are key in determining the fate of a cell upon TNF stimulation. In the absence of LUBAC, TNF‐mediated signalling is shifted from NF‐κB activation towards induction of cell death via complex‐II (Haas *et al*, 2009; Tokunaga *et al*, 2009; Gerlach *et al*, 2011; Ikeda *et al*, 2011; Peltzer *et al*, 2014, 2018). Mouse experiments have shown that the resulting TNF‐induced cell death can be highly inflammatory as genetic ablation of LUBAC components leads to multi‐organ inflammation (HogenEsch *et al*, 1993; Seymour *et al*, 2007; Gerlach *et al*, 2011; Ikeda *et al*, 2011; Tokunaga *et al*, 2011), in large part caused by TNF‐induced cell death (Kumari *et al*, 2014; Rickard *et al*, 2014). Importantly, homozygous mutations in the LUBAC components HOIP and HOIL‐1 have been identified in patients suffering from chronic autoinflammatory syndromes (Boisson *et al*, 2012, 2015).

Deubiquitinases (DUBs) are proteases that cleave Ub modifications and functionally revert Ub signalling. Several DUBs, including A20 and CYLD, act as negative regulators of NF‐κB signalling to balance activation and ensure resolution of inflammation (Harhaj & Dixit, 2012). OTULIN (OTU family deubiquitinase with linear Ub specificity, also known as FAM105B or Gumby) is the only DUB known to specifically cleave M1 linkages (Keusekotten *et al*, 2013; Rivkin *et al*, 2013). Several studies have shown that OTULIN binds directly to the LUBAC subunit HOIP (Elliott *et al*, 2014; Schaeffer *et al*, 2014; Takiuchi *et al*, 2014). In the absence of OTULIN, M1‐linked Ub chains accumulate in cells and receptor signalling complexes, enhancing NF‐κB activation in response to stimulation of TNF‐R1 and NOD2 (Fiil *et al*, 2013; Keusekotten *et al*, 2013; Rivkin *et al*, 2013; Draber *et al*, 2015; Damgaard *et al*, 2016; Hrdinka *et al*, 2016; van Wijk *et al*, 2017). Interestingly, similar to LUBAC components, homozygous mutations in *OTULIN* were recently found to cause autoinflammation in humans (Damgaard *et al*, 2016; Zhou *et al*, 2016), showing that excessive M1‐linked Ub signalling can be harmful. OTULIN‐related autoinflammatory syndrome (ORAS) (also known as otulipenia or autoinflammation, panniculitis and dermatosis syndrome (AIPDS); OMIM ID: 617099) is a potentially fatal, TNF‐driven autoinflammatory disease characterised by sterile systemic inflammation, recurrent high fevers, panniculitis, diarrhoea, arthritis and general failure to thrive (Damgaard *et al*, 2016; Zhou *et al*, 2016). Key features of ORAS are recapitulated in OTULIN‐deficient mice (Damgaard *et al*, 2016). However, the cellular defects underlying the development of inflammation in ORAS remain unclear.

Here, we report a new case of ORAS identified by whole‐exome sequencing of a patient with neonatal‐onset, severe inflammatory symptoms. Primary fibroblast cultures from this patient reveal a surprising phenotype of deregulated M1‐linked Ub signalling, whereby OTULIN loss coincides with LUBAC downregulation to prevent intrinsic NF‐κB activation. In contrast, OTULIN‐deficient myeloid cells are hyper‐inflammatory and spontaneously activate NF‐κB and secrete TNF. Both cell types, however, are sensitised to cell death in response to TNF. Importantly, we find that haematopoietic stem cell transplantation (HSCT) leads to complete resolution of all inflammatory symptoms associated with ORAS. This shows that haematopoietic cells are crucial for the clinical manifestation of ORAS and indicates HSCT could be are curative treatment for the disease.

## Results

### Inflammatory symptoms in a patient with homozygous *OTULIN* mutations

**Figure 1:**
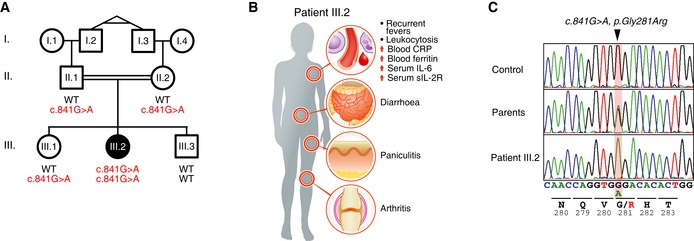
Mutations in OTULIN in a new case of OTULIN‐related autoinflammatory syndrome (ORAS) ASegregation of the inflammatory symptoms (filled symbols) and the c.841G>A substitution in OTULIN in the affected kindred. ○, females; □, males; double lines, consanguineous relationship. Probands I.2 and I.3 are monozygotic twins. Roman numerals indicate generations.BSchematic representation of the symptoms and clinical presentation of patient III.2.COTULIN DNA sequence chromatograms showing the homozygous single base substitution (*c.841G>A, p.Gly281Arg*, arrowhead). Data are representative to two independent experiments.

A female patient of Arab origin (patient III.2), the second of three children born to first‐degree related parents (her grandfathers are identical twins; Fig 1A), developed severe inflammatory symptoms shortly after birth. From the age of 3 days, she developed severe idiopathic, systemic inflammation and had recurrent episodes of high fever in combination with widespread panniculitis (Fig 1B and Appendix Clinical Description). At the age of 7 months, her symptoms included high fevers, diarrhoea and panniculitis, and she was cachectic, weighing 3.4 kg (< 3^rd^ percentile; WHO Multicentre Growth Reference Study Group, 2006) and had severe splenomegaly and bilateral cataracts. Laboratory evaluation revealed elevated acute phase proteins, including C‐reactive protein (CRP) and ferritin, elevated IL‐6 and soluble IL‐2 receptor (sIL‐2R) in serum, severe anaemia, and leukocytosis with significant monocytosis in the absence of any evidence of infection (Fig 1B and Appendix Clinical Description).

The consanguineous relations in the family (Fig 1A) indicated that patient III.2 suffered from an autosomal recessive disorder. Genetic testing excluded Mediterranean fever and chronic atypical neutrophilic dermatosis with lipodystrophy and elevated temperature (CANDLE) syndrome caused by mutations in *MEFV* and *PSMB8*, respectively. Whole‐exome sequencing (WES) was performed to investigate patient III.2's underlying genetic defect. Filtering out all common and heterozygous variants, WES revealed a single, homozygous G‐to‐A substitution in *OTULIN*,* c.841G>A; p.Gly281Arg,* in patient III.2 (Figs 1A and C and Appendix Table S1). The parents of patient III.2 (II.1 and II.2) and her sister (III.1) were heterozygous for the substitution, whereas her brother (III.3) did not carry the mutation (Figs 1A and C). WES revealed no other homozygous or previously annotated pathogenic variants likely to cause the disease phenotype (Appendix Table S1).

Mutations in *OTULIN* have recently been described to cause ORAS, an autosomal recessive autoinflammatory disease (Damgaard *et al*, 2016; Zhou *et al*, 2016). Patient III.2's symptoms, as well as the course of disease and effect of treatments (see below, Appendix Clinical Description), resembled those of other ORAS patients (Damgaard *et al*, 2016; Zhou *et al*, 2016). Hence, patient III.2 was diagnosed with ORAS.

### Mutation of Gly281 to Arg reduces OTULIN's activity towards M1‐linked Ub by unfolding the substrate‐binding site

To date, three different mutations in OTULIN have been reported in families with patients affected by ORAS. In one family, a single nucleotide deletion introduces a premature stop codon, Gly174Aspfs*2, leading to deletion of roughly two‐thirds of the catalytic OTU domain (Zhou *et al*, 2016) and thus likely a complete loss of function although the N‐terminal fragment may be present in cells. Hypomorphic Leu272Pro and Tyr244Cys missense mutations were identified in two other families (Damgaard *et al*, 2016; Zhou *et al*, 2016). Leu272 is located in the Ub‐binding pocket in the OTU domain and the mutation to Pro impairs M1‐linked diUb binding, catalytic efficiency and stability of OTULIN (Damgaard *et al*, 2016). Tyr244 is not found or predicted to make any contacts to Ub or be involved in catalysis (Keusekotten *et al*, 2013), and the main effect of the Tyr244Cys mutation might be reduced protein stability (Zhou *et al*, 2016).

**Figure EV1:**
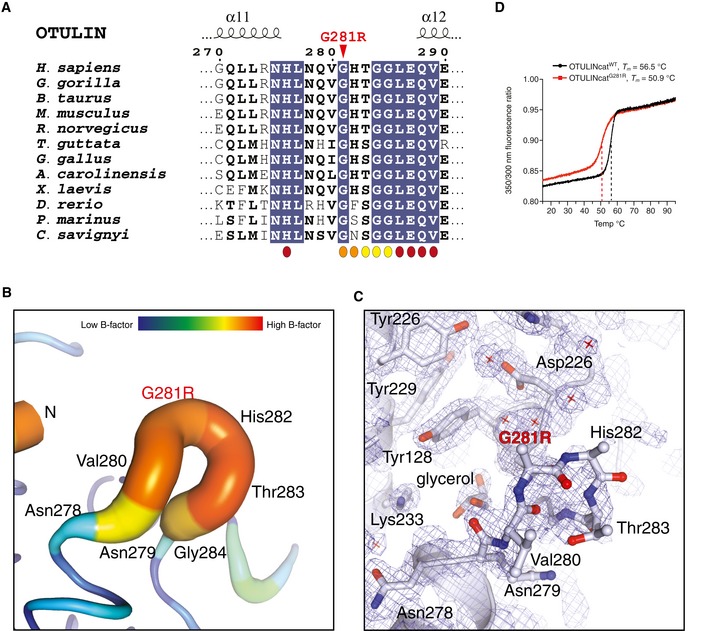
Structural analysis of OTULIN^G^ ^281R^ (related to Fig 2) AMultiple sequence alignment of the sequence surrounding Gly281 in OTULIN's catalytic domain. The Gly281Arg mutation is indicated by a red arrowhead. Residues interacting with the proximal (orange), distal (maroon) or both Ub (yellow) moieties are indicated.BPutty representation of the α11‐α12 loop incorporating the Gly281Arg mutation from the OTULIN^G281R^ structure (PDB: 6I9C). An increased radius of the cartoon and increase in colours yellow‐red represent increased crystallographic temperature, B factors, and reflect the increased disorder of the α11‐α12 loop relative to the rest of the structure (comparable B factors are only found at the N and C termini where there is no secondary structure).CElectron density for the same region as in (B). A weighted 2Fo‐Fc map is shown contoured at 1σ. Residues from the α11‐α12 loop are shown as stick representation. Owing to the increased motility of the α11‐α12 loop, no interpretable electron density was observed for the side chains of Gly218Arg and His282 and only the Cα and backbone amide could be confidently fitted into the electron density map. Other residues in the region had interpretable electron density and several water molecules in the region were sufficiently ordered to be modelled (red cross).DTryptophan fluorescence upon thermal unfolding of OTULINcat^WT^ and OTULINcat^G281R^ (1.0 mg/mL) measured by nanodifferential scanning fluorimetry (nano‐DSF). Apparent melting temperatures (*T*_*m*_) are indicated (dashed lines). Data are representative to two independent experiments.

**Figure 2:**
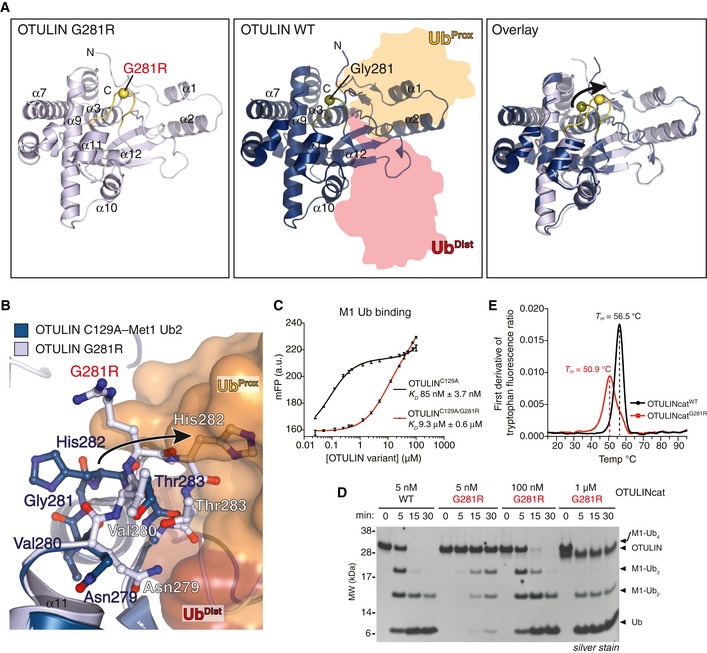
Mutation of Gly281 to Arg unfolds OTULIN's substrate‐binding site and reduces its catalytic activity AOverall structure of OTULIN^G281R^ (left, PDB: 6I9C) and OTULIN^C129A^ bound to M1‐linked diUb (middle, PDB: 3ZNZ; Keusekotten *et al*, 2013) and a superimposition of the two structures (right). The Cα positions of Gly281/Arg281 are shown as spheres.BClose‐up view of the M1‐linked Ub‐binding site from OTULIN^G281R^ (light blue) and OTULIN's catalytic domain (blue) with M1‐linked diUb bound (proximal Ub, orange; distal Ub, red) showing a clash between proximal Ub and His282 upon G281R mutation (arrow).CAffinity measurements by fluorescence polarisation (FP) with recombinant catalytically inactive OTULIN^C129A^ or OTULIN^C129A/G281R^ and FlAsH‐labelled M1‐linked diUb. Data represent mean ± SD of one experiment performed in technical triplicate. Data are representative of three independent experiments. a.u., arbitrary units. *K*_*D*_, dissociation constant.DM1‐linked tetraUb hydrolysis by recombinant OTULIN^WT^ and OTULIN^G281R^ using the indicated OTULIN concentrations and visualised on silver‐stained SDS–PAGE. Data are representative of three independent experiments.EFirst derivative of tryptophan fluorescence upon thermal unfolding of recombinant OTULINcat^WT^ and OTULINcat^G281R^ (1.0 mg/ml) measured by nanodifferential scanning fluorimetry (nano‐DSF). Apparent melting temperatures (*T*_*m*_) are indicated (dashed lines). Data are representative to two independent experiments.

Similar to Leu272 and Tyr244, Gly281 is also located in the OTU domain (Keusekotten *et al*, 2013). Gly281 is evolutionarily conserved and located in a loop between helices α11 and α12 (Fig EV1A; Keusekotten *et al*, 2013). Together with His282 and Thr283, Gly281 forms part of the Ub‐binding site and makes direct contacts to the proximal Ub of M1‐linked diUb bound across the active site (Figs 2A, centre panel and EV1A; Keusekotten *et al*, 2013). While Gly281 is in an important region, the effect of the mutation to Arg is difficult to predict. To understand the pathogenicity of the Gly281Arg mutation on OTULIN, we crystallised OTULIN's catalytic domain (OTULINcat, aa 80–352) containing the Gly281Arg ORAS mutation (OTULINcat^G281R^) and determined its structure to 1.8 Å resolution (Figs 2A and Appendix Table S2). Overall, the structure showed that OTULINcat^G281R^ adopted an OTU fold similar to OTULINcat^WT^ (Keusekotten *et al*, 2013) without any major perturbations (Fig 2A). However, comparing the structure of OTULINcat^G281R^ with the previously solved structure of OTULINcat in complex with M1‐linked diUb (Keusekotten *et al*, 2013) showed that the Gly281Arg mutation partly unfolds the substrate‐binding site (Fig 2A, right panel). The introduced Arg side chain can no longer fit into the narrow pocket between helices α3 and α9 thereby flipping the Gly281‐containing loop out of its normal binding groove (Figs 2A and B; Keusekotten *et al*, 2013). In the mutant protein, the loop has increased flexibility, as represented by the high crystallographic B‐factor distribution across the loop compared to other regions within the OTU domain (Fig EV1B) and also evidenced by lack of interpretable electron density for the side chains of Val280, mutated Arg281 and His282 (Fig EV1C). The new conformation of the Gly281‐containing loop indicated that His282 and Thr283 would clash with M1‐linked diUb at the binding site and prevent substrate binding (Fig 2B).

We probed the binding of M1‐linked diUb to catalytically inactive OTULIN^C129A^ or OTULIN^C129A/G281R^. As indicated from the structure, the Gly281Arg mutation strongly reduced binding of M1‐linked diUb to OTULIN and the dissociation constant (*K*_*D*_) of OTULIN^C129A/G281R^ and M1‐linked diUb was 9.3 μM compared with 85 nM in OTULIN^C129A^ (Fig 2C).

The reduced substrate binding by OTULIN^G281R^ was also reflected in catalytic activity. OTULIN^G281R^ was ~20‐200 times less active towards M1‐linked tetraUb compared with OTULIN^WT^ (Fig 2D). At a 5 nM concentration, OTULIN^WT^ cleaved ~50% of the M1‐linked tetraUb intro tri‐, di‐ and mono‐Ub within 5 min and all tetraUb was processed at 15 min. In contrast, between 100 nM and 1 μM of OTULIN^G281R^ was needed to achieve the same catalytic efficiency (Fig 2D).

In addition to reduced Ub binding and activity, the Gly281Arg mutation caused a considerable intrinsic destabilisation of OTULIN. Monitoring protein unfolding over a thermal gradient, we observed that the melting temperature (*T*_*m*_) was reduced from 56.5°C for OTULIN^WT^ to 50.9°C for OTULIN^G281R^ (Figs 2E and EV1D). This intrinsic loss of stability indicates a defect in folding of the mutant. Such defects can be exacerbated in cells where partially damaged or unfolded proteins trigger protein quality control mechanisms, including protein degradation.

### M1‐linked Ub accumulation and destabilisation of OTULIN^G281R^ and LUBAC in patient fibroblasts

**Figure 3:**
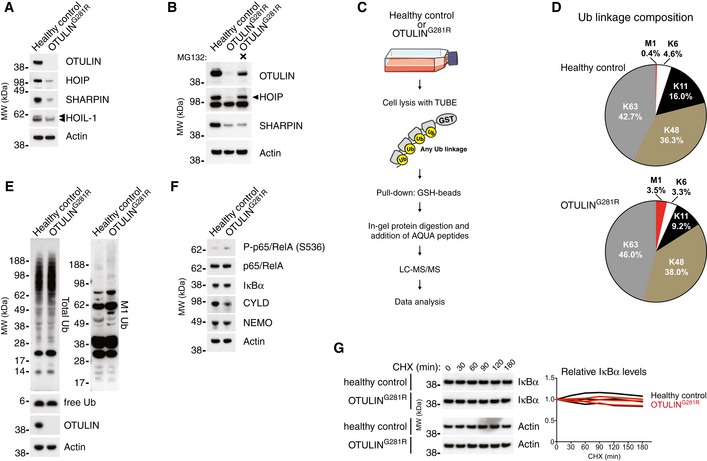
LUBAC degradation and accumulation of M1‐linked Ub in OTULIN^G^ ^281R^ fibroblasts AImmunoblot analysis of whole‐cell lysates from untreated primary healthy control and patient fibroblasts. Data are representative of three independent experiments.BImmunoblot analysis of whole‐cell lysates from primary healthy control and patient fibroblasts either left untreated or treated with the proteasome inhibitor MG132 (10 μM) for 24 h. Data are representative of two independent experiments.CSchematic representation of the AQUA‐MS/MS‐based proteomics approach for quantification of cellular Ub linkage composition.DAQUA‐MS/MS data from TUBE‐based purification of cellular polyUb conjugates from untreated primary fibroblasts from a healthy control or patient III.2 harbouring the OTULIN^G281R^ mutation. K27, K29, linkages could not be detected, and K33 could not be accurately quantified in all samples. Data are representative of two independent experiments (see Fig EV2F and G).E, FImmunoblot analysis of whole‐cell lysates from untreated primary healthy control or patient fibroblasts. Data are representative of three independent experiments.GImmunoblot (left) and densitometry (right) analysis of IκBα levels in primary healthy control and patient fibroblasts treated with cycloheximide (CHX) (50 μg/ml) as indicated. Data are representative of three independent experiments. Source data are available online for this figure.

**Figure EV2:**
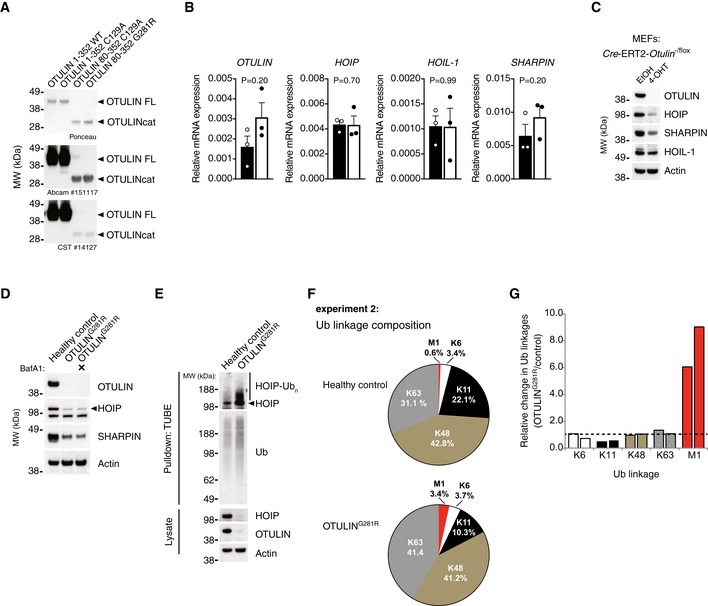
Analysis of protein and mRNA expression in OTULIN^G^ ^281R^ and quantitative proteomics analysis of the cellular Ub linkage composition (related to Fig 3) AAnalysis of antibody recognition of recombinant OTULIN proteins. 200 ng recombinant protein was loaded in each lane. Membranes were stained with Ponceau S before immunoblot analysis was performed with the two anti‐OTULIN antibodies used in this study (#14127 from Cell Signaling Technology and ab151117 from Abcam). Data are representative of two independent experiments.BRelative mRNA levels of *OTULIN, HOIP, HOIL‐1 and SHARPIN* from primary healthy control and patient fibroblasts measured by quantitative RT–PCR. Bars represent mean ± SEM of three independent experiments each performed in duplicate. Statistical significance was determined using the Mann–Whitney *U*‐test.CImmunoblot analysis of whole‐cell lysates from Cre‐ERT2‐*Otulin* ^−/flox^ inducible knock‐out MEFs treated with 4‐hydroxytamoxifen (4‐OHT) (100 nM) or ethanol (EtOH) alone as indicated. Data are representative of two independent experiments.DImmunoblot analysis whole‐cell lysates from primary healthy control and patient fibroblasts either left untreated or treated with the autophagosome inhibitor bafilomycin A1 (BafA) (100 nM) for 24 h. Data are representative of two independent experiments.EImmunoblot analysis of endogenous Ub conjugates purified by TUBE pull‐down from untreated healthy control and patient fibroblasts shows HOIP ubiquitination in the patient cells. Data are representative of two independent experiments.FBiological replicate of the experiment shown in Fig 3D. AQUA‐MS/MS data from TUBE‐based purification of cellular polyUb conjugates from untreated primary fibroblasts from a healthy control or patient III.2 harbouring the OTULIN^G281R^ mutation. K27, K29, linkages could not be detected and K33 could not be accurately quantified in all samples.GRelative change (OTULIN^G281R^/control) in the amount of cellular Ub linkages. Bars represent the data presented in Figs 3D and EV2F individually. Source data are available online for this figure.

The cellular defects underlying the development of inflammation in ORAS are unclear. To study how OTULIN mutations impact on non‐haematopoietic cells and how these cells may contribute to the clinical aspects of ORAS, e.g. panniculitis, we established a primary dermal fibroblast culture from patient III.2. Surprisingly, immunoblot analysis showed that OTULIN^G281R^ was virtually undetectable in the patient cells (Fig 3A), suggesting that the partial unfolding of OTULIN^G281R^ observed *in vitro* (Fig 2E) indeed destabilises the protein. The Gly281Arg mutation did not affect detection of OTULIN by the antibodies used in this study, which both recognise OTULIN's N terminus (Fig EV2A), supporting the notion that OTULIN^G281R^ is destabilised in cells. Treatment with the proteasome inhibitor MG132 substantially increased OTULIN^G281R^ levels (Fig 3B), and *OTULIN* transcript levels remained similar between healthy control and ORAS fibroblasts (Fig EV2B), strongly indicating that the reduced OTULIN^G281R^ level is caused by proteasomal degradation.

Interestingly, we observed a substantial concomitant destabilisation of the LUBAC components HOIP and SHARPIN, whereas HOIL‐1 remained largely stable except for a reduction in the level of a slower‐migrating form (HOIL‐1L) (Fig 3A). We have previously reported a similar loss of LUBAC in murine B and T cells (Damgaard *et al*, 2016), and we also observed a loss of LUBAC in mouse embryonic fibroblasts (MEFs) with induced OTULIN deletion (Fig EV2C), showing this phenomenon is not restricted to human fibroblasts. Like for murine T and B cells (Damgaard *et al*, 2016), transcript levels of *HOIP* (also known as *RNF31*), *HOIL‐1* (also known as *RBCK1*) and *SHARPIN* were largely unaltered between OTULIN^G281R^ fibroblasts and controls (Fig EV2B) and inhibition of autophagosomal degradation could not increase LUBAC protein levels (Fig EV2D). Instead, we found that HOIP was polyubiquitinated in the OTULIN^G281R^ fibroblasts (Fig EV2E) and that HOIP levels could be rescued almost completely by proteasomal inhibition (Fig 3B), consistent with proteasome‐mediated degradation of LUBAC, particularly HOIP, in the absence of OTULIN in the patient fibroblasts.

To assess how the reduction in OTULIN, HOIP and SHARPIN levels in OTULIN^G281R^ fibroblast impacted the Ub system and the cellular composition of Ub chain types, we used a non‐selective tandem Ub‐binding entity (TUBE; Hjerpe *et al*, 2009) to purify polyUb chains from cells in a non‐linkage‐selective manner (Fig 3C). The polyUb chains purified by the TUBE were analysed by absolute quantification (AQUA) tandem mass spectrometry (MS/MS; Kirkpatrick *et al*, 2006; Fig 3C). K6, K48 and K63 linkages combined comprised ~80–85% of the cellular polyUb chains and did not change, while K27, K29 and K33 linkages were undetectable or unquantifiable in the OTULIN^G281R^ and control fibroblasts (Figs 3D and EV2F and G). Curiously, we noted a reduction in K11 linkages from ~19% in healthy control fibroblasts to ~10% in OTULIN^G281R^ cells (Figs 3D and EV2F and G). Immunoblotting showed no apparent overall differences in the total levels of Ub conjugates or free Ub in whole‐cell lysates between the patient fibroblasts and healthy control cells (Fig 3E).

Strikingly, despite the reduction in LUBAC levels, the AQUA‐MS/MS analysis revealed a considerable increase in M1‐linked Ub chains from ~0.5% in healthy controls to ~3.5% in OTULIN^G281R^ fibroblasts (Figs 3D and EV2F and G). This implies that the remaining LUBAC is still active and may function unantagonised. The increase in cellular M1 Ub levels, however, was hardly detectable by immunoblotting, and only a very faint increase in the high molecular weight smear was observed in the OTULIN^G281R^ fibroblasts (Fig 3E). This indicates that the increase in M1 linkages might be relatively low compared to the levels observed in OTULIN‐deficient cells that retain LUBAC expression (Draber *et al*, 2015; Damgaard *et al*, 2016; van Wijk *et al*, 2017). Consistently, OTULIN^G281R^ fibroblasts exhibited no appreciable signs of spontaneous p65/RelA phosphorylation or IκBα degradation (Fig 3F and G). This suggests that, in the absence of OTULIN, LUBAC is downregulated via proteasomal degradation in the patient fibroblasts, potentially to reduce the levels of M1‐linked Ub and prevent intrinsic activation of NF‐κB signalling.

### Signalling from the TNF‐RSC is impaired in OTULIN^G281R^ fibroblasts

**Figure 4:**
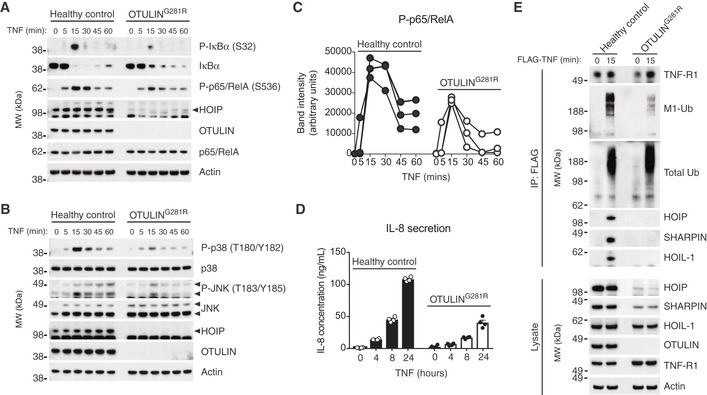
TNF‐induced NF‐κB and MAP kinase activation is impaired in OTULIN^G^ ^281R^ fibroblasts due to reduced LUBAC recruitment to the TNF‐RSC AImmunoblot analysis of IκBα phosphorylation and degradation as well as p65/RelA phosphorylation in healthy control or OTULIN^G281R^ fibroblasts primary fibroblasts in response to stimulation with TNF (10 ng/ml). Data are representative of three independent experiments.BImmunoblot analysis of phosphorylation of the MAP kinases p38 and JNK in healthy control or OTULIN^G281R^ primary fibroblasts in response to stimulation with TNF (10 ng/ml). Data are representative of three independent experiments.CDensitometry analysis of p65/RelA phosphorylation as presented in (A) from three independent experiments.DELISA analysis of IL‐8 secretion in response to TNF stimulation (10 ng/ml) in healthy control or OTULIN^G281R^ primary fibroblasts. Bars represent mean ± SEM (*n *=* *4).EImmunoblot analysis of the native TNF‐RSC purified by immunoprecipitation from healthy control or OTULIN^G281R^ primary fibroblasts. Data are representative of three independent experiments. Source data are available online for this figure.

Decreased OTULIN function increases M1‐linked Ub conjugation and NF‐κB activation in response to TNF stimulation in LUBAC‐proficient cells (Fiil *et al*, 2013; Keusekotten *et al*, 2013; Damgaard *et al*, 2016; Hrdinka *et al*, 2016). Yet, it is unknown how ORAS cells with concomitant reduction of HOIP and SHARPIN levels respond to TNF‐R1 engagement. We treated the primary OTULIN^G281R^ patient fibroblasts with TNF and analysed NF‐κB and MAP kinase activation by immunoblotting. Interestingly, in contrast to other OTULIN‐deficient cells or cells with RNAi‐mediated knock‐down of OTULIN (Fiil *et al*, 2013; Keusekotten *et al*, 2013; Damgaard *et al*, 2016; Hrdinka *et al*, 2016), TNF signalling was decreased in OTULIN^G281R^ fibroblasts, although not completely abrogated (Fig 4A–C), similar to the response of LUBAC‐deficient cells (Haas *et al*, 2009; Gerlach *et al*, 2011; Ikeda *et al*, 2011; Tokunaga *et al*, 2011; Peltzer *et al*, 2014). Stimulation with TNF led to decreased phosphorylation and degradation of IκBα as well as reduced phosphorylation of p65/RelA compared with healthy controls (Fig 4A and C). Similarly, phosphorylation and activation of the MAP kinases p38 and JNK was impaired in the OTULIN^G281R^ fibroblasts (Fig 4B). This correlated with a substantial reduction in IL‐8 secretion from the patient cells in response to TNF (Fig 4D), showing that reduced NF‐κB and MAP kinase signalling leads to functional impairment of innate immune signalling in the ORAS fibroblasts in response to TNF.

After stimulation of TNF‐R1, LUBAC translocates to the TNF‐RSC and conjugates M1‐linked Ub chains to multiple components of the complex (Gerlach *et al*, 2011; Draber *et al*, 2015). We immunoprecipitated the native TNF‐RSC after stimulation with TNF, and indeed all three LUBAC components, HOIP, SHARPIN and HOIL‐1, together with high molecular weight M1‐linked Ub conjugates, co‐precipitated with TNF‐R1 from healthy control fibroblasts (Fig 4E). However, in the OTULIN^G281R^ fibroblasts, recruitment of LUBAC to TNF‐R1 was strongly reduced and hardly detectable (Fig 4E). Interestingly, also HOIL‐1, which remains relatively stable in the patient fibroblasts, could hardly be detected at TNF‐RSC, further confirming that HOIL‐1 and SHARPIN are recruited in a HOIP‐dependent manner (Draber *et al*, 2015). Consistently, the amount of M1‐linked Ub conjugates, but not total Ub conjugates, at the TNF‐RSC was drastically reduced in the patient cells (Fig 4E). Collectively, this suggests that the primary effect of OTULIN loss in the patient fibroblasts, namely the degradation of LUBAC components to prevent intrinsic signalling and autoactivation, results in a secondary effect: the failure to mount a sufficient LUBAC‐dependent response to TNF stimulation.

### NF‐κB activation and TNF secretion in human OTULIN‐deficient monocytes

**Figure 5:**
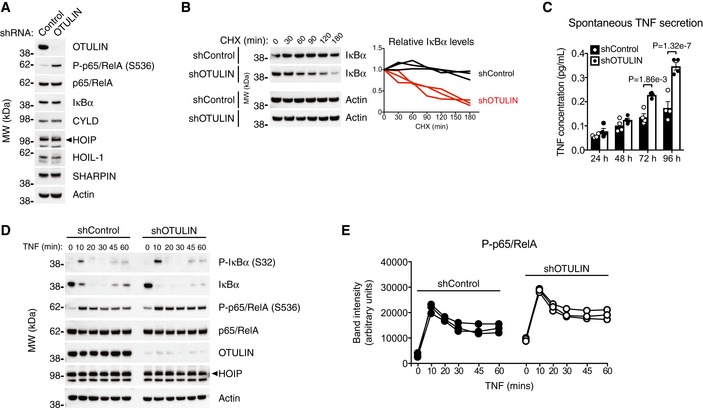
OTULIN deficiency in human THP‐1 monocytes leads to increased NF‐κB activation and spontaneous TNF secretion AImmunoblot analysis of whole‐cell lysate from untreated human THP‐1 monocytes with stable expression of a non‐targeting control shRNA or and shRNA targeting OTULIN. Data are representative of two independent experiments.BImmunoblot (left) and densitometry (right) analysis of IκBα levels in shControl and shOTULIN THP‐1 cells treated with cycloheximide (CHX) (50 μg/ml) as indicated. Data are representative of three independent experiments.CELISA analysis of spontaneous TNF secretion over 96 h in shControl and shOTULIN THP‐1 cells. Data represent mean ± SEM (*n *=* *4) and were analysed using the two‐way ANOVA test of statistical significance with Sidak's correction for multiple comparisons.DImmunoblot analysis of IκBα phosphorylation and degradation as well as p65/RelA phosphorylation in shControl and shOTULIN THP‐1 cells in response to stimulation with TNF (10 ng/ml). Data are representative of three independent experiments.EDensitometry analysis of p65/RelA phosphorylation as presented in (D) from three independent experiments. Source data are available online for this figure.

Conditional deletion of OTULIN in myeloid cells recapitulates many aspects of ORAS in mice (Damgaard *et al*, 2016). This is associated with increased NF‐κB activation and spontaneous TNF secretion from OTULIN‐deficient BMDMs, which, in contrast to T cells, B cells, MEFs and ORAS fibroblasts, retain normal LUBAC levels after loss of OTULIN (Damgaard *et al*, 2016; Figs 3A and EV2C). Yet, how human myeloid cells react to loss of OTULIN is unclear. To assess this, we analysed NF‐κB activation and TNF secretion in human THP‐1 monocytes with stable, short‐hairpin RNA (shRNA)‐mediated knock‐down of OTULIN (shOTULIN cells) compared with cells expressing a non‐targeting shRNA (shControl cell) (Hrdinka *et al*, 2016). The shOTULIN THP‐1 cells showed substantial reduction in cellular OTULIN levels, although some OTULIN remained (Fig 5A). Similar to OTULIN‐deficient murine BMDMs, LUBAC remained stable in shOTULIN cells and the levels of HOIP, HOIL‐1 and SHARPIN were indistinguishable from those of shControl cells (Fig 5A). Importantly, OTULIN deficiency in THP‐1 monocytes led to spontaneous phosphorylation of p65/RelA (Fig 5A) and drastically increased turnover of IκBα (Fig 5B), indicative of NF‐κB activation in shOTULIN cells. Significantly, shOTULIN cells spontaneously secreted TNF in the absence of any stimulus (Fig 5C). This shows that the shOTULIN cells have increased basal NF‐κB activation and spontaneously secrete cytokines, similar to our previous report for OTULIN‐deficient BMDMs (Damgaard *et al*, 2016).

As the shOTULIN cells spontaneously secrete TNF, we examined their signalling after TNF stimulation. In response to TNF, the shOTULIN cells also exhibited a stronger NF‐κB signalling response (Fig 5D). Immunoblotting revealed increased IκBα phosphorylation and degradation as well as elevated p65/RelA phosphorylation after TNF stimulation in the shOTULIN cells compared with shControls (Figs 5D and E).

This shows that also human OTULIN‐deficient myeloid cells have a TNF‐associated hyper‐inflammatory phenotype and indicates that myeloid cells may be fundamentally different from fibroblasts in their response to loss or reduction of OTULIN function.

### OTULIN deficiency sensitises fibroblasts and monocytes to TNF‐induced cell death

**Figure 6:**
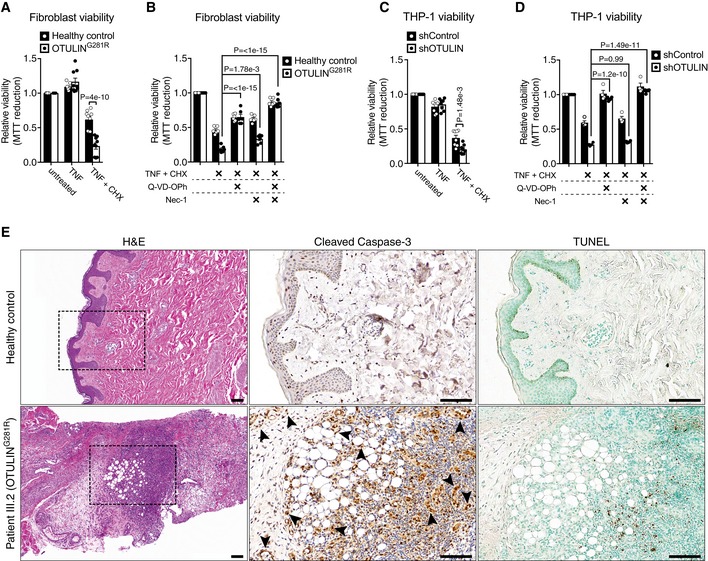
OTULIN deficiency sensitises cells to TNF‐induced apoptosis A–DViability of healthy control or OTULIN^G281R^ primary fibroblasts (A, B) and shControl and shOTULIN THP‐1 cells (C, D) after 24 h and 6 h, respectively, of treatment with TNF (100 ng/ml), CHX (50 μg/ml), Q‐VD‐OPh (10 μM) and Nec‐1 (10 μM) as indicated was analysed using MTT reduction assays. Results were normalised to untreated samples. Each experiment was performed in duplicate. Bars represent mean ± SEM (A, *n *=* *9; B, *n *=* *6; C, *n *=* *8; D, *n *=* *4) and were analysed using the two‐way ANOVA test of statistical significance with Sidak's correction for multiple comparisons.EIncreased apoptotic cell death in the skin of ORAS patient III.2. Serial sections of normal skin (top panels) and a skin biopsy from patient III.2 taken at an inflammatory flare (bottom panels) were immunostained for cleaved caspase‐3 (centre panels) or analysed by terminal deoxynucleotidyl transferase dUTP nick end labelling (TUNEL) assay (right panels). Arrowheads indicate cleaved caspase‐3‐positive mesenchymal cells. None of these markers were present in the healthy control skin. H&E, haematoxylin and eosin. Boxes in H&E panels indicate the areas magnified in the cleaved caspase‐3 and TUNEL panels. Scale bars, 100 μm. Data are representative of stained sections from three healthy controls and one biopsy from ORAS patient III.2.

**Figure EV3:**
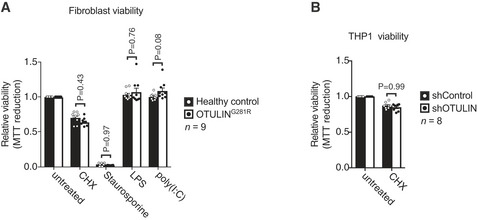
Analysis of viability of healthy control or OTULIN^G^ ^281R^ primary fibroblasts and shControl or shOTULIN THP‐1 cells (related to Fig 6) AViability of healthy control or OTULIN^G281R^ primary fibroblasts after 24 h of treatment with CHX (50 μg/ml), staurosporine (1 μM), LPS (100 ng/ml) or poly(I:C) (1 μg/ml) as indicated was analysed using MTT reduction assays. Results were normalised to untreated samples. Each experiment was performed in duplicate. Bars represent mean ± SEM (*n *=* *9) and were analysed using the two‐way ANOVA test of statistical significance with Sidak's correction for multiple comparisons.BViability of healthy control or shControl and shOTULIN THP‐1 cells after 6 h of combination treatment with TNF (100 ng/ml) and CHX (50 μg/mL) as indicated was analysed using MTT reduction assays. Results were normalised to untreated samples. Each experiment was performed in duplicate. Bars represent mean ± SEM (*n *=* *8) and were analysed using the two‐way ANOVA test of statistical significance with Sidak's correction for multiple comparisons.

Dysregulation of TNF‐induced NF‐κB activation in various tissues and cell types can result in inflammation due to increased cell death (Pasparakis, 2009). The autoinflammatory conditions caused by LUBAC deficiency are examples of this. SHARPIN‐deficient *cpdm* (chronic proliferative dermatitis) mice (HogenEsch *et al*, 1993; Seymour *et al*, 2007; Gerlach *et al*, 2011; Ikeda *et al*, 2011) and patients with mutations in *HOIP* or *HOIL‐1* (Boisson et al, 2012, 2015, 2015) develop severe autoinflammatory syndromes. LUBAC destabilisation caused by these genetic alterations impairs TNF signalling and NF‐κB activation (Gerlach *et al*, 2011; Ikeda *et al*, 2011; Tokunaga *et al*, 2011; Boisson et al, 2012, 2015), leading to TNF‐R1‐dependent induction of (mainly) TRADD‐, FADD‐ and caspase‐8‐dependent apoptosis (Gerlach *et al*, 2011; Ikeda *et al*, 2011; Peltzer *et al*, 2014, 2018), which is the main driver of inflammation (Kumari *et al*, 2014; Rickard *et al*, 2014). Additionally, knock‐in mice expressing catalytically inactive OTULIN^C129A^ die mid‐gestation (~E10.5) due to aberrant cell death (Heger *et al*, 2018), similar to HOIP‐ or HOIL‐1‐deficient mice (Peltzer *et al*, 2014, 2018). We tested whether OTULIN^G281R^ ORAS fibroblasts were sensitised to TNF‐induced cell death. Interestingly, we found that OTULIN^G281R^ fibroblasts, despite their reduction in LUBAC levels, were not sensitive to cell death induced by TNF (Fig 6A) or a range of other stimuli (Fig EV3A). This is in contrast to LUBAC‐deficient cells (Gerlach *et al*, 2011; Ikeda *et al*, 2011; Peltzer *et al*, 2014, 2018) or cells expressing inactive OTULIN^C129A^ (Heger *et al*, 2018), which are all sensitive to cell death induced by TNF alone. We found, however, that the OTULIN^G281R^ fibroblasts were sensitive to cell death induced by treatment with TNF in combination with the protein synthesis inhibitor cycloheximide (CHX; Fig 6A), suggesting that under certain stress conditions the patient fibroblasts are sensitised to TNF‐induced cell death.

TNF‐induced cell death may occur either via caspase‐dependent apoptosis or RIPK1 kinase‐dependent necroptosis (Fuchs & Steller, 2015). Treatment of the OTULIN^G281R^ fibroblasts with the general caspase inhibitor Q‐VD‐OPh or the RIPK1 kinase inhibitor necrostatin‐1 (Nec‐1) showed that caspase inhibition rescued the majority of the TNF+CHX‐induced cell death in the ORAS fibroblasts (Fig 6B). Nec‐1 treatment alone only conferred mild protection against cell death, but treatment of ORAS fibroblasts with a combination of Q‐VD‐OPh and Nec‐1 led to an almost complete rescue (Fig 6B). This shows that the OTULIN^G281R^ fibroblasts likely die by apoptosis and to a lesser extent by necroptosis, consistent with the mode of TNF‐induced cell death observed in LUBAC‐deficient cells in culture and in SHARPIN‐deficient *cpdm* mice (Gerlach *et al*, 2011; Ikeda *et al*, 2011; Berger *et al*, 2014; Kumari *et al*, 2014; Rickard *et al*, 2014).

Interestingly, despite their normal LUBAC levels, the shOTULIN THP‐1 cells were also sensitised to TNF+CHX‐induced cell death, but not to cell death by TNF alone (Figs 6C and EV3B), similar to the OTULIN^G281R^ fibroblasts. The cell death in shOTULIN cells could completely be prevented by treatment with Q‐VD‐Oph, and Nec‐1 treatment had no significant effect, neither alone nor in combination with caspase inhibition (Fig 6D). This is consistent with a recent report showing that LUBAC, in addition to its pro‐survival role at TNF‐R1, also contributes to TNF‐induced apoptosis by inducing cFLIP degradation (Tang *et al*, 2018).

Inactivation of OTULIN by expression of OTULIN^C129A^ in adult mice has recently been reported to cause systemic inflammation, which can be prevented by combined ablation of cell death pathways by deletion of caspase‐8, RIPK3 and RIPK1 (Heger *et al*, 2018). Together with the increased cell death we observe *in vitro*, this suggests that cell death may contribute to ORAS pathogenesis. We analysed skin biopsies from ORAS patient III.2, taken at an inflammatory flare, and healthy control subjects for markers of cell death. Skin from the ORAS patient showed evidence of prominent inflammation with diffuse neutrophil infiltration, particularly around adipocytes, in the dermis and subcutaneous tissues, consistent with panniculitis (Fig 6E and Appendix Clinical Description). This inflammation correlated with the presence of cleaved caspase‐3‐positive and TUNEL‐positive cells in the dermis and subcutaneous tissues (Fig 6E), showing that cell death occurs *in vivo* during ORAS inflammation. Intriguingly, cleaved caspase‐3‐positive cells appeared to be mainly mesenchymal cells, including fibroblasts (Fig 6E, centre panels, arrowheads), whereas TUNEL‐positive cells appeared to be mainly infiltrating leukocytes (Fig 6E, bottom panels).

These data show that cell death (including apoptosis) occurs in ORAS and that it may contribute to the TNF‐driven pathogenesis of the disease.

### Haematopoietic stem cell transplantation ameliorates ORAS

**Figure 7:**
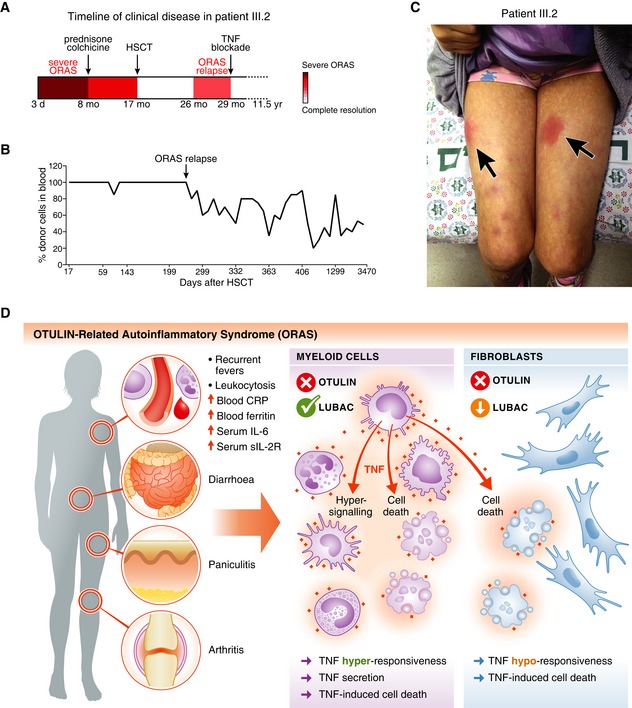
HSCT ameliorates clinical ORAS symptoms in patient III.2 ASchematic of the timeline of clinical disease in patient III.2 (see Appendix Clinical Description). Arrows indicate therapeutic interventions. d, day; mo, month; yr, year.BAnalysis of the blood cell chimerism in patient III.2 after HSCT. Short terminal repeat (STR) analysis was used to determine the percentage of donor cells in peripheral blood. Arrow indicates point of relapse.CPhotograph of patient III.2 at age ˜10 years at an episode of inflammation caused by delayed etanercept administration. Arrows indicate the erythematous subcutaneous nodules (panniculitis).DModel of the cellular effects of OTULIN deficiency in myeloid cells and fibroblasts in ORAS. Hyper‐signalling and TNF secretion in myeloid cells as well as TNF‐induced cell death of both haematopoietic cells and cell types with LUBAC downregulation, e.g. dermal fibroblasts, may contribute to the inflammation, pathogenesis and clinical manifestation of ORAS.

Previously, we have shown that deletion of OTULIN in all haematopoietic cell types in mice, or in myeloid cells alone, is sufficient to recapitulate key features of ORAS (Damgaard *et al*, 2016). But whether haematopoietic cells are necessary or sufficient to drive ORAS in humans is unknown. Importantly, we could use the case of ORAS in patient III.2 to address this retrospectively. As outlined, patient III.2 developed severe inflammatory symptoms within the first months of her life and she had a poor response to conventional anti‐inflammatory treatment (Appendix Clinical Description). From the age of 8 months, she was treated with prednisone and colchicine, to which she only had a partial response (Fig 7A), similar to previous reports from ORAS patients (Damgaard *et al*, 2016; Zhou *et al*, 2016). At the age of 11 months, the recombinant IL‐1 receptor antagonist anakinra was added to the treatment regimen without any improvement of her symptoms. Because of this poor response to treatment and the impaired pre‐transplant performance status of the patient, HSCT was performed at the age of 17 months (Fig 7A) with reduced intensity fludarabine‐based conditioning that included fludarabine (30 mg/m^2^/day for 6 days), busulphan (3.2 mg/kg/day for 4 days) and thiotepa (10 mg/kg/day for 1 day) together with anti‐thymocyte globulin (ATG) (10 mg/kg). The donor was the patient's HLA‐matched father. The course of transplantation was uneventful, and 100% donor chimerism was achieved 17 days after HSCT (Fig 7B and Appendix Clinical Description). Remarkably, HSCT resulted in complete resolution of all inflammatory symptoms, including panniculitis, arthritis and diarrhoea, and the patient experienced complete clinical remission (Fig 7A and Appendix Clinical Description). As we and others have found signalling and cell death defects in OTULIN‐deficient non‐haematopoietic cells (Figs 3 and 4; Heger *et al*, 2018), complete remission after HSCT strikingly shows that OTULIN deficiency in haematopoietic cells is necessary for clinical manifestation of ORAS including the organ‐specific symptoms panniculitis and diarrhoea (Fig 7D).

Nine months after HSCT, at the age of 26 months, patient III.2 relapsed with fevers, panniculitis and arthritis, albeit with less severe symptoms than before the transplantation (Fig 7A and C, and Appendix Clinical Description). The relapse correlated with a progressive decrease in bone marrow chimerism and regrowth of patient III.2's haematopoietic cells (Fig 7B). In mice, bone marrow chimerism with 50% OTULIN‐deficient cells is sufficient to cause severe systemic ORAS‐like inflammation (Damgaard *et al*, 2016), indicating that the decrease in chimerism observed in patient III.2 (Fig 7B) is sufficient to cause the relapse.

After the relapse, treatment with prednisone was resumed until the age of 29 months where patient III.2 started treatment with the TNF‐blocking agent etanercept (soluble TNF receptor fusion protein; 0.4 mg/kg, twice weekly; Fig 7A and Appendix Clinical Description). This led to immediate and complete resolution of her inflammatory symptoms, and corticosteroid treatment was discontinued. Now, at age ~12 years, patient III.2 is treated with low dose etanercept (0.8 mg/kg) every 10 days and she remains asymptomatic as long as administration is regular (Fig 7C and Appendix Clinical Description).

Collectively, these data show that haematopoietic cells are required for clinical manifestation of ORAS and confirm that TNF is the driver cytokine for all inflammatory aspects of the disease (Damgaard *et al*, 2016), adding ORAS to the growing list of TNF‐related pathologies (Kalliolias & Ivashkiv, 2016; Manthiram *et al*, 2017). Intriguingly, the dependence on OTULIN‐deficient haematopoietic cells for ORAS implies that myeloid cells are the main drivers of the TNF‐mediated inflammation (Figs 5 and 7D), as OTULIN deficiency in B or T cells alone in mice does not produce any inflammatory phenotypes (Damgaard *et al*, 2016). Moreover, it indicates that cell death in ORAS might be a key contributing and amplifying result of TNF‐mediated inflammation (Figs 6E and 7D).

## Discussion

Here, we report a new case of ORAS caused by homozygous hypomorphic *c.841G>A; p.Gly281Arg* mutations in a patient from a consanguineous family. To date, only three families (five patients) with *OTULIN* mutations and ORAS manifestations had been reported (Damgaard *et al*, 2016; Zhou *et al*, 2016). Our report of a new case of ORAS caused by a novel Gly281Arg mutation adds a new family and patient that expands our understanding of the pathogenesis of the disease, its clinical aspects, as well as its treatment.

Correct regulation of M1‐linked Ub conjugation by LUBAC and hydrolysis by OTULIN is critical to maintain immunehomeostasis in humans and mice. This is evident from clinical genetic studies (Boisson et al, 2012, 2015; Damgaard *et al*, 2016; Zhou *et al*, 2016) and numerous mouse studies (reviewed in Hrdinka & Gyrd‐Hansen, 2017). Interestingly, the clinical result of mutations in *HOIL‐1* or *HOIP* (i.e. reduced M1‐linked Ub conjugation) or in *OTULIN* (i.e. reduced M1‐linked Ub hydrolysis) is the same: inflammation. Clinically, HOIL‐1 and OTULIN deficiency manifests similarly with neonatal‐onset systemic inflammation, recurrent fevers and failure to thrive (Boisson *et al*, 2012; Damgaard *et al*, 2016; Zhou *et al*, 2016). However, some important differences between these diseases exist. HOIL‐1 deficiency is associated with severe primary immunodeficiency, cardiomyopathy and muscle weakness (Boisson *et al*, 2012; Nilsson *et al*, 2013; Wang *et al*, 2013), of which there is no evidence in ORAS patients (Damgaard *et al*, 2016; Zhou *et al*, 2016). In addition, TNF blockade is highly effective in all ORAS patients reported. Standard immunosuppression (e.g. corticosteroids) is, on the other hand, generally ineffective (Damgaard *et al*, 2016; Zhou *et al*, 2016). In contrast, corticosteroid treatment is generally effective in HOIL‐1 deficiency whereas anti‐TNF treatment in these patients is not (Boisson *et al*, 2012). This suggests that the underlying defects causing these syndromes, while overlapping, may be different.

OTULIN‐related autoinflammatory syndrome patients respond poorly to standard anti‐inflammatory and immunosuppressive treatments (Damgaard *et al*, 2016; Zhou *et al*, 2016), but their inflammatory symptoms can be alleviated by neutralisation of TNF (Damgaard *et al*, 2016; Zhou *et al*, 2016). These treatments, however, have to be regular (and likely lifelong) as omission or delay in administration can cause inflammation to flare‐up (Fig 7C and Appendix Clinical Description). Currently, there is no cure for ORAS. We provide here the first report of the outcome of HSCT for ORAS. Remarkably, HSCT led to complete remission of the patient's severe ORAS symptoms, including panniculitis, arthritis and diarrhoea, showing that haematopoietic cells are crucial for manifestation of the disease. The patient's unfortunate relapse appears to be due to regrowth of her haematopoietic cells (Fig 7B). The decreased donor chimerism is most likely a consequence of the reduced intensity pre‐transplantation conditioning, which may have enabled proliferation of residual haematopoietic stem cells in the patient, leading to a drop in donor chimerism and a relapse of inflammatory symptoms. We therefore suggest the use of more intense, fully myeloablative conditioning regimes as preparation for HSCT if such regimes can be tolerated by the recipients. Despite the patient's relapse, this case supports further studies into HSCT as a potential treatment to ameliorate ORAS.

The Gly281Arg mutation identified in this study has a drastic impact on OTULIN. Not only does it reduce binding of and catalytic activity towards M1‐linked Ub chains, but it also destabilises the protein such that OTULIN^G281R^ is hardly detectable in the absence of proteasome inhibitor in patient fibroblasts. Destabilisation of OTULIN by ORAS mutations has been described for two other point mutations, Leu272Pro and Tyr244Cys, as well as the premature stop mutant Gly174Aspfs*2 (Damgaard *et al*, 2016; Zhou *et al*, 2016). The combination of reduced catalytic activity and very low protein levels of OTULIN^G281R^ in cells indicates that the mutation might cause close to a complete loss of OTULIN function *in vivo*. The effect of the Gly281Arg mutation on OTULIN is similar in mechanism to the Leu272Pro mutation identified in a different ORAS family (Damgaard *et al*, 2016; Zhou *et al*, 2016). Collectively, this strongly suggests that ORAS is a disease of loss or significant reduction of OTULIN activity and expression.

In cells, we observed a considerable increase in M1 Ub linkages in the OTULIN^G281R^ patient fibroblasts, despite the drastically reduced HOIP and SHARPIN levels. At steady state, the cellular levels of M1‐linked Ub are very low (Figs 3D and EV2F) (Fiil *et al*, 2013; Keusekotten *et al*, 2013; Draber *et al*, 2015), yet they accumulate transiently in receptor complexes to facilitate signalling (Fig 4E) (Gerlach *et al*, 2011; Emmerich *et al*, 2013; Fiil *et al*, 2013; Keusekotten *et al*, 2013; Peltzer *et al*, 2014; Rickard *et al*, 2014; Draber *et al*, 2015; Hrdinka *et al*, 2016; Kupka *et al*, 2016a). This suggests that M1‐linked Ub is a potent and highly regulated signal that is strictly controlled in cells to prevent intrinsic activation or dysregulation of signalling. In the OTULIN^G281R^ fibroblasts, we observed a chronic and global accumulation of M1‐linked Ub chains (Figs 3D and EV2F and G) together with a striking concomitant reduction in LUBAC levels and no evidence of spontaneous NF‐κB activation (Figs 3A, F and G). This suggests that in certain cell types, e.g. T and B cells (Damgaard *et al*, 2016) and fibroblasts (Figs 3A and EV2C), the potentially uncontrolled M1‐linked Ub accumulation in the absence of OTULIN may function as a “stress” signal that somehow feeds back and reduces LUBAC activity by downregulating the level of this complex via the proteasome, thereby maintaining homeostasis by preventing or reducing intrinsic activation of inflammatory signalling. However, downregulation of LUBAC also means that these cells cannot respond appropriately to external signals, including TNF, which may in turn contribute to ORAS pathogenesis (Fig 7D, see below).

Different cell types respond differently to loss of OTULIN. In murine models, LUBAC is stable in OTULIN‐deficient myeloid cells, but not in B and T cells where HOIP and SHARPIN levels are reduced (Damgaard *et al*, 2016). Reduction in LUBAC levels, primarily in HOIP and SHARPIN, has also been reported in fibroblasts from three different ORAS patients (Zhou *et al*, 2016). Similarly, we observe here that HOIP and SHARPIN levels are reduced in OTULIN^G281R^ fibroblasts while LUBAC remains stable in OTULIN‐deficient THP‐1 monocytes. This striking cell type‐specific effect on LUBAC suggests that the phenotype of different cell types upon loss of OTULIN may depend on their LUBAC status. LUBAC exists, at least, as a trimeric HOIP‐HOIL‐1‐SHARPIN complex (Gerlach *et al*, 2011; Ikeda *et al*, 2011; Tokunaga *et al*, 2011), but the stoichiometry of components and whether it varies between cell types is unknown. Specific subpopulations of LUBAC, e.g. dimeric HOIP‐HOIL‐1 or HOIP‐SHARPIN complexes or complexes stably associated with other factors such as DUBs, remain a topic of active investigation. Recently, it was reported that OTULIN and CYLD, via its adaptor SPATA2, utilise the same mechanism to interact with HOIP, thereby forming mutually exclusive complexes (Draber *et al*, 2015; Elliott *et al*, 2016). This suggests that distinct pools of LUBAC‐DUB complexes may exist (Hrdinka & Gyrd‐Hansen, 2017). The apparently specific downregulation of HOIP and SHARPIN in ORAS patient fibroblasts (Fig 3A; Zhou *et al*, 2016) and in T and B cells (Damgaard *et al*, 2016) might suggest that OTULIN could be a part of and/or regulate a distinct HOIP‐SHARPIN complex. However, this needs further investigation. Our data suggest that careful assessment of steady state levels of OTULIN and LUBAC, and possibly CYLD, is necessary for appropriate interpretation of experiments involving OTULIN, LUBAC and M1‐linked Ub signalling.

OTULIN^G281R^ fibroblasts are functionally LUBAC‐deficient after TNF stimulation. LUBAC recruitment and M1‐linked Ub conjugation at the TNF‐RSC are reduced, accompanied by impaired TNF signalling, decreased IL‐8 secretion and sensitisation to TNF‐induced cell death in the presence of CHX (Fig 4). Interestingly, OTULIN‐deficient THP‐1 cells, which are hyper‐inflammatory and hyper‐signal in response to TNF (Fig 6), are also sensitive to this form of cell death, despite their normal levels of LUBAC. These observations suggest that ORAS may not only be caused by TNF‐induced hyper‐signalling and cytokine secretion (Damgaard *et al*, 2016), but that ORAS pathogenesis may also involve cell death (Fig 7D). Our analysis of cell death in biopsies from ORAS patient III.2 supports this notion and provides clear evidence of apoptosis in the skin of this patient at the time of an inflammatory flare. This bears resemblance to the dermatitis phenotypes in LUBAC‐deficient mice and the cell death observed in LUBAC‐deficient cells (Kumari *et al*, 2014; Peltzer *et al*, 2014, 2018; Rickard *et al*, 2014; Taraborrelli *et al*, 2018). However, in contrast to LUBAC deficiency where cells are sensitised to cell death induced by TNF alone, OTULIN‐deficient cells need additional perturbation (i.e. CHX) before they become sensitive to TNF‐induced death. This could suggest that cell death of OTULIN‐deficient cells may be a secondary effect of a “stressed” environment, such as an inflamed tissue. The dermatitis in SHARPIN‐deficient *cpdm* mice is caused by cell or tissue intrinsic defects leading to TNF‐induced cell death of keratinocytes (Kumari *et al*, 2014; Rickard *et al*, 2014) and it is not mediated by immune cells as allogeneic BM or splenocyte transplantations fail to transfer the disease to WT mice (HogenEsch *et al*, 1993; Rickard *et al*, 2014). In contrast, OTULIN deficiency in haematopoietic cells is necessary for manifestation of skin inflammation/panniculitis in ORAS patients as demonstrated here by HSCT (Fig 7A–C and Appendix Clinical Description). Yet, mouse models indicate that OTULIN deficiency in haematopoietic cells is not sufficient for development of the full spectrum of ORAS symptoms. Mice with genetic ablation of OTULIN either in all haematopoietic cell types or specifically in myeloid cells develop systemic ORAS‐like inflammation, but the organ‐specific symptoms that are hallmarks of the human disease (panniculitis, diarrhoea and arthritis) do not manifest spontaneously in these models (Damgaard *et al*, 2016). The full clinical disease in ORAS may therefore be a “compound” phenotype where hyper‐inflammatory, TNF‐secreting haematopoietic (myeloid) cells cause TNF‐induced inflammation and cell death of both non‐haematopoietic cells with LUBAC downregulation, e.g. dermal fibroblasts, and haematopoietic cells (Fig 7D), thereby giving rise the organ‐specific hallmark symptoms panniculitis, diarrhoea and arthritis.

Together, these clinical and experimental data expand our understanding of the cellular mechanisms leading to ORAS development and indicate HSCT as a new potential treatment to ameliorate ORAS.

## Materials and Methods

### Whole‐exome analysis

Exonic sequences from a DNA sample from patient III.2 were enriched using SureSelect Human All Exon 50 Mb V.5 Kit (Agilent Technologies, Santa Clara, CA) and sequenced on an Illumina HiSeq2500 (Illumina, San Diego, CA) with 100‐bp paired‐end reads. Data were analysed on the DNAnexus platform (DNAnexus, Inc., Mountain View, CA) using default parameters with the human genome assembly hg19 (GRCh37) as reference. For further details, see Appendix Supplementary Methods.

### Expression and purification of recombinant proteins

OTULIN and TUBE proteins were purified from *E. coli* as described in (Elliott *et al*, 2014) and (Hrdinka *et al*, 2016), respectively. For further details, see Appendix Supplementary Methods.

### Qualitative DUB assay

Qualitative Ub cleavage assays were performed as previously described (Keusekotten *et al*, 2013). For further details, see Appendix Supplementary Methods.

### Protein crystallisation and structure determination

OTULINcat^G281R^ crystals were grown by hanging drop vapour diffusion method and the structure solved by molecular replacement using PHASER (McCoy *et al*, 2007) with wild‐type OTULINcat (PDB ID 3ZNV) as the search model. For further details, see Appendix Supplementary Methods.

### Binding assays

Fluorescence anisotropy experiments were performed as previously described (Keusekotten *et al*, 2013). For further details, see Appendix Supplementary Methods.

### Differential scanning fluorimetry (DSF) thermal unfolding experiments

Nano‐DSF measurements were performed as previously described (Damgaard *et al*, 2016). For further details, see Appendix Supplementary Methods.

### Cell culture

A primary fibroblast culture from patient III.2 was established from a skin punch biopsy. Primary fibroblasts were cultured in Ham's F‐10 nutrient mix + GlutaMAX™ (Life Technologies) supplemented with 20% iron‐supplemented foetal calf serum (FCS) (#C8056, Sigma) (v/v) and penicillin/streptomycin (Life Technologies). Human THP‐1 monocytes with stable short‐hairpin RNA (shRNA)‐mediated knock‐down of OTULIN (shOTULIN) or a non‐targeting control (shControl) were a kind gift from Dr. Mads Gyrd‐Hansen (University of Oxford) (Hrdinka *et al*, 2016). THP‐1 cells were cultured undifferentiated in RPMI‐1640 + GlutaMAX™ (Life Technologies) supplemented with 1 mM sodium pyruvate (Life Technologies), 10% FCS (Life Technologies) (v/v), 50 μM β‐mercaptoethanol and penicillin/streptomycin (Life Technologies). All cells were kept at 37°C in a humidified atmosphere at 5% CO_2_. All cultures were found to be negative for mycoplasma contamination by regular PCR testing (Young *et al*, 2010).

### Cell stimulations and signalling experiments

Primary fibroblasts or THP‐1 cells were treated as indicated in figures and legends. For signalling experiments, cells were stimulated with recombinant human TNF (Life Technologies). For proteasomal inhibition, cells were treated with 10 μM MG132 (Sigma) in DMSO as indicated. For inhibition of autophagosomal degradation, cells were treated with 100 nM bafilomycin A1 from *Streptomyces griseus* (BafA) (Sigma) in DMSO as indicated. Cells were lysed either directly in sample buffer (50 mM Tris pH 6.8, 10% glycerol (v/v), 100 mM DTT, 2% SDS (w/v), bromophenol blue), sonicated using a microtip for 5 s at 30% amplitude on a Vibra‐Cell™ VC750 (Sonics & Materials, Inc., Newton, CT) and boiled for 2 min, or lysed in RIPA buffer (50 mM Tris pH 7.4, 1% NP‐40 (v/v), 0.5% deoxycholate (w/v), 0.1% SDS (w/v), 150 mM NaCl, 2 mM EDTA, 5 mM MgCl_2_) for 20 min on ice, lysate cleared by centrifugation and supplemented with 4× sample buffer. For assessment of IκBα stability, THP‐1 cells were washed in PBS and seeded in 96‐well plates in complete medium ~16 h before they were treated with 50 μg/ml cycloheximide (CHX; Santa Cruz Biotechnology, Santa Cruz, CA) in DMSO as indicated. IκBα stability in primary fibroblasts was assayed by seeding the fibroblasts in 12‐well plates 18–24 h prior to treatment with CHX as indicated. After CHX treatment, all cells were washed in PBS and lysed in RIPA buffer.

### Immunoprecipitation of the TNF‐RSC

Immunoprecipitation of the TNF receptor signalling complex (TNF‐RSC) was performed using 100 ng/ml FLAG‐tagged TNF (Enzo Life Sciences, Farmingdale, NY) as previously described (Fiil *et al*, 2013). For further details, see Appendix Supplementary Methods.

### Purification of endogenous polyUb conjugates by TUBE pull‐down

Endogenous polyUb conjugates were purified from primary fibroblasts using TUBE affinity reagents as described previously (Damgaard *et al*, 2012; Fiil *et al*, 2013). For further details, see Appendix Supplementary Methods.

### Ub chain composition analysis (AQUA‐MS)

Details on LC‐MS/MS methods for analysis of the cellular composition of Ub chain linkages can be found in Appendix Supplementary Methods.

### Immunoblotting

Samples were resolved on 4–12% Bis‐Tris NuPAGE gels (Life Technologies) and transferred to nitrocellulose or polyvinylidene fluoride (PVDF) membranes. Blots were visualised using Clarity™ Western ECL Substrate (Bio‐Rad) on a ChemiDoc™ Touch or ChemiDoc™ MP imager (Bio‐Rad). All antibodies are listed in Appendix Table S3. Densitometry analysis of immunoblots was performed using the Fiji software (Schindelin *et al*, 2012). For further details, see Appendix Supplementary Methods.

### ELISA

Cytokine and chemokine concentrations in cell culture medium were measured using the Human IL‐8/CXCL8 Quantikine ELISA kit (D8000C) or the Human TNF‐alpha Quantikine HS ELISA kit (HSTA00E) from R&D Systems, Minneapolis, MN, according to the manufacturer's instructions. For IL‐8 measurements, equal numbers of primary fibroblasts from the OTULIN^G281R^ patient or healthy control were seeded in flat‐bottom 96‐well cell culture plates 24 h before the experiments. Cells were washed, and fresh culture medium was added immediately before the cells were treated with 10 ng/ml recombinant human TNF (Life Technologies) as indicated. The cell culture medium was collected at the indicated times (for the “0” samples, medium from untreated cells was left on the cells for 24 h and collected at the same time at the 24 h TNF‐treated samples) and subjected to ELISA analysis. For TNF measurements, equal numbers of shControl and shOTULIN THP‐1 cells were washed in PBS and seeded in fresh medium in round‐bottom 96‐well plates. The cells were then left untreated, and the culture medium was collected at the indicated times and subjected to ELISA analysis.

### MTT reduction assay for cell viability

MTT reduction assay was performed as previously described (Damgaard *et al*, 2013) with cells treated with recombinant human TNF (100 ng/ml; Life Technologies), ultrapure LPS from *E. coli* K12 (100 ng/ml; Invivogen), poly(I:C) (1 μg/ml; Invivogen), staurosporine (1 μM; Sigma), cycloheximide (CHX; 50 μg/ml; Santa Cruz Biotechnology), or a combination of recombinant human TNF and CHX with or without the caspase inhibitor Q‐VD‐OPh (10 μM; BioVision, Milpitas, CA) or the RIPK1 kinase inhibitor necrostatin‐1 (Nec‐1) (10 μM; Cayman Chemical, Ann Arbor, MI) as indicated. For further details, see Appendix Supplementary Methods.

### Histology, immunohistochemistry and TUNEL assay

Biopsies were fixed in formalin and embedded in paraffin before sectioning and staining. Biopsies from patient III.2 were obtained with informed consent (see below) from the pathological archives at Hadassah Hebrew University Medical Center, Jerusalem, Israel. Healthy skin control biopsies were obtained from the Human Research Tissue Bank, Cambridge Biomedical Research Centre, Cambridge University Hospitals, UK. Deparaffinisation, haematoxylin and eosin (H&E) staining, cleaved caspase‐3 immunohistochemical staining (anti‐cleaved caspase‐3, #9661, RRID AB_2341188, Cell Signaling Technology) with haematoxylin counterstaining, and TUNEL assays (TdT *In Situ* Apoptosis Detection kit, #4810‐30‐K, R&D Systems) with methyl green counterstaining were performed by NDBbio Laboratories, LLC., Baltimore, MD. Stained slides were scanned (20× objective), and image files (SVS files) were processed using the QuPath software (https://qupath.github.io/; Bankhead *et al*, 2017).

### Statistics

Data are presented as mean ± standard error of the mean (SEM) or ± standard deviation (SD) as indicated. Sample number (*n*) indicates the number of independent biological samples in each experiment. Sample numbers and experimental repeats are indicated in figures and figure legends. Data were analysed using the two‐way analysis of variance (ANOVA) with Sidak's correction for multiple comparisons or the non‐parametric Mann–Whitney *U*‐test of the null hypothesis as indicated. For the two‐way ANOVA tests, the Shapiro–Wilk test was used to assess normality of the data distributions. Generally, the datasets were normally distributed and as the variables analysed (cytokine secretion and cell viability) are generally accepted to be normally distributed, the use of the two‐way ANOVA test was considered appropriate. Differences in means were considered statistically significant at *P* < 0.05. Analyses were performed using the GraphPad Prism software version 7.0b.

### Study approval

Written informed consent was obtained from all subjects and family members. The study was approved by the ethical committees of Hadassah Medical Center and the Ministry of Health, Israel (Y.B), and the South Birmingham Research Ethics Committee, UK (E.R.M.), and performed in accordance with the ethical standards laid down in the 1964 Declaration of Helsinki.

For additional methods and details, see Appendix Supplementary Methods.

**The paper explained**

**Problem**

OTULIN is a deubiquitinase that removes methionine 1 (M1)‐linked polyubiquitin chains conjugated by the linear ubiquitin chain assembly complex (LUBAC), and mutations OTULIN cause OTULIN‐related autoinflammatory syndrome (ORAS), a TNF‐driven autoinflammatory disease in humans. ORAS manifests clinically with a range of neonatal‐onset inflammatory symptoms, including fever, skin inflammation (panniculitis), diarrhoea and arthritis. It was unclear which cells types are responsible for the development of these symptoms and how OTULIN deficiency affects M1‐linked polyubiquitin signalling in cells to cause inflammation.

**Results**

We identified a new case of ORAS in which a Gly281 to Arg mutation in OTULIN causes the disease. To assess the cellular impact of loss of OTULIN in ORAS, we analysed the inflammatory signalling response in patient‐derived fibroblasts and in OTULIN‐deficient human monocytes. This revealed that OTULIN loss in patient‐derived fibroblasts leads to concomitant reduction of LUBAC and decreased TNF signalling, whereas OTULIN‐deficient monocytes retain LUBAC expression and show increased signalling and TNF secretion. However, both cell types are sensitised to TNF‐induced cell death. Remarkably, haematopoietic stem cell transplantation leads to complete resolution of all inflammatory symptoms associated with ORAS, showing that haematopoietic cells are necessary for development of ORAS inflammation. This indicates that ORAS pathogenesis involves hyper‐inflammatory immune cells and TNF‐induced death of both immune cells and non‐haematopoietic cells.

**Impact**

OTULIN‐related autoinflammatory syndrome is a potentially fatal, neonatal‐onset autoinflammatory disease of unclear pathogenesis. Treatment with TNF inhibitors such as antibodies (e.g. infliximab) or TNF blockers (e.g. etanercept) effectively ameliorates inflammation, but there is currently no cure for ORAS. We show that haematopoietic cells are necessary for clinical manifestation of ORAS, suggesting that haematopoietic stem cell transplantation is a potentially curative treatment for ORAS.

## Author contributions

RBD, OE, DK and YB conceived the project and coordinated the research. RBD, PRE and KNS designed experiments. RBD performed and analysed all cell culture experiments, including the primary patient cells. PRE performed and analysed the biochemical and biophysical experiments. KNS performed and analysed the mass spectrometry experiments. RBD, PRE, KNS and DK interpreted the data. ERM provided experimental support and access to the Human Research Tissue Bank. YB, OE and PS were responsible for human clinical and genetic studies. RBD and DK wrote the paper with input from all authors.

## For more information

https://www.omim.org/entry/617099

## Supporting information



Appendix

Expanded View Figures PDF

Source Data for Expanded View

Review Process File

Source Data for Figure 3

Source Data for Figure 4

Source Data for Figure 5

## Data Availability

Due to restrictions from the patient consent approved by the research ethics committees, it is not be possible to deposit complete exome sequencing data in a public repository, but the data could be made available to interested researchers by contacting the corresponding author (Y.B.). Accession numbers, coordinates and structure factors for crystal structures of OTULIN^G281R^ have been deposited within the protein data bank with accession code 6I9C. Mass spectrometry data include global ubiquitin linkage analysis by AQUA and been deposited to the Mass Spectrometry Interactive Virtual Environment (MassIVE) (ftp://massive.ucsd.edu/MSV000083154) at University of California San Diego, CA.
