# Supplementary material for: OTULIN deficiency in ORAS causes cell type‐specific LUBAC degradation, dysregulated TNF signalling and cell death
Source: EMBO Mol Med. 2019 Feb 25;11(3):e9324. doi: 10.15252/emmm.201809324 (PMC6404114; doi:10.15252/emmm.201809324)
Supplement: Supplementary file 1 — Appendix [file EMMM-11-e9324-s001.pdf]

APPENDIX

OTULIN deficiency in ORAS causes LUBAC degradation, dysregulated TNF signalling, and cell death

Rune B. Damgaard, Paul R. Elliott, Kirby N. Swatek, Eamonn R. Maher, Polina Stepensky, Orly Elpeleg, David Komander, Yackov Berkun

Table of contents:

Appendix Clinical Description

Appendix Supplementary Methods

Appendix Table S1: Genetic variants identified by WES

Appendix Table S2: Data collection refinement statistics

Appendix Table S3: Primary and secondary antibodies

Supplementary References

Appendix Clinical Description

The consultand is a 11.5-year-old girl, second of three children of first-degree related parents (grandfathers are identical twins) of Arab origin. She was delivered prematurely at 36 weeks of uneventful gestation by normal vaginal delivery; her birth weight was 1.89 kg. At the age of 3 days, due to cyanosis, she underwent evaluation and was treated for neonatal sepsis. Several erythematous subcutaneous nodules were noticed on the lower extremities.

At age of 2.5 months she was admitted to another hospital with fever, multiple skin nodules and severe normocytic anaemia (Hb 6.5 gr %). She was referred to our hospital at age of 7 months, following recurrent episodes of high fever, diarrhoea, erythematous skin lesions and red eyes, lasting for 3-7 days. On presentation, she appeared ill and cachectic, weighting 3.4 kg. She had conjunctival injection and bilateral cataracts, erythematous tender subcutaneous nodules, severe splenomegaly and red mildly swollen fingers. The evaluation revealed elevated acute phase reactants during the usual attack: leucocytosis with significant (up to 40%) monocytosis; elevated blood levels CRP 5-10 mg% (<0.5); high ferritin 200-300 ng/ml, and elevated serum levels of IL-6 and soluble IL-2 receptor (sIL-2R). She was negative for ANA, and she had normal levels of IgM, IgA, IgE, and IgG as well as complement C3 and C3 for her age. Immunophenotyping demonstrated normal T, B and NK cell subgroups, count, and function and normal oxidative burst and function of polymorphonuclear cells. Skin biopsy showed deep dermis and subcutaneous tissue with dense diffuse neutrophil infiltrates with relatively increased numbers of eosinophils, findings compatible with sterile suppurative panniculitis. The epidermis was normal with mild spongiosis, without findings compatible with vasculitis of cutaneous blood vessels. Biopsy that was performed between inflammatory attacks showed mild, mainly intraseptal panniculitis. Bone marrow biopsy was normal except for mildly increased number of megakaryocytes and mature leucocytes. Diagnosis of undefined autoinflammatory disorder was raised and therapy with prednisone and colchicine was initiated at age of 8 months, with a partial response. At age of 11-month interleukin-1 (IL-1) receptor antagonist anakinra was added without any response.

At age of 17 months, because of the poor response to the conventional treatment, haematopoietic stem cell transplantation (HSCT) was performed. The donor was the patient's fully HLA-matched father. Conditioning consisted fludarabine (30 mg/m^2 for six days), busulphan (3.2 mg/kg for four days), thiotepa (10 mg/kg) and anti-thymocyte globulin (ATG) (10 mg/kg). She was successfully engrafted 17 days after transplantation and 100% donor chimerism was achieved. This led to complete resolution of all inflammatory symptoms and attacks.

Shortly after HSCT, chronic GVHD, mainly of skin, appeared. Nine months after HSCT, progressive decrease in chimerism was noted, accompanied by relapse of less severe inflammatory attacks that included, in addition to fever and panniculitis of extremities, also arthritis, mainly of small joints. Donor leukocyte infusion (DLI) was tried without any improvement in her BM chimerism. The patient was retreated with corticosteroids and anakinra, could not lead to a decrease of corticosteroid dose. At age of 29 months, blockade of tumour necrosis factor (TNF) by a soluble TNF-receptor fusion protein etanercept, 0.4 mg/kg twice weekly, lead to immediate resolution of the inflammatory symptoms and attacks, enabling discontinuation of corticosteroids. At age of 4 years, due to severe bilateral cataracts, lensectomy was performed. Recently, the 11.5-year-old patient is asymptomatic on low dose etanercept (0.8 mg/kg given every 10 days). Omissions of a single injection or delay in administration has led to the usual inflammatory attack, including high fever, arthritis of small joints, mainly fingers, and red tender skin nodules, usually 12-14 days after the last etanercept injection.

Genetic testing of the patient found no pathogenic mutations in *MEFV* (Mediterranean fever) and *PSMB8* (proteasome subunit beta type-8), excluding Mediterranean Fever and CANDLE syndrome as the cause of inflammation.

Given the consanguinity in this family, we performed exome analysis under the hypothesis of a recessively inherited, rare, causal allele. Whole exome sequence analysis yielded 44.5 million mapped reads with a mean coverage of 69X. Following alignment and variant calling, we performed a series of filtering steps. These included removing variants which were called less than 8X, were off-target, heterozygous, synonymous, had MAF>1% at ExAC (Exome Aggregation Consortium, Cambridge,

MA, (URL: http://exac.broadinstitute.org)) or MAF>2% at the Hadassah in-house database (~1200 ethnic matched exome analyses). 16 homozygous variants, which resided in disease associated genes, survived the filtering (Table EV1), but using conservation score and phenotypic relevance we focused on the homozygous variant Hg19 *Chr5:14690394 G>A*, *p.Gly281Arg* in the *OTULIN* gene (also known as *FAM105B* or *Gumby*). This variant was confirmed by Sanger sequencing. The parents and sister were heterozygous for the mutation and the brother was homozygous for the WT allele. Furthermore the variant was not carried by any of the over 120,000 healthy individuals whose exomes were deposited in gnomAD website (http://gnomad.broadinstitute.org). Thus, the patient was diagnosed with OTULIN-Related Autoinflammatory Syndrome (ORAS).

Appendix Supplementary Methods

Whole exome analysis

Exonic sequences from a DNA sample from patient III.2 were enriched using SureSelect Human All Exon 50 Mb V.5 Kit (Agilent Technologies, Santa Clara, CA), and parallel DNA sequencing was undertaken on an Illumina HiSeq2500 (Illumina, San Diego, CA) with 100bp paired-end reads. This yielded 44.5 million mapped reads with 69X mean coverage. Read alignment and variant calling were performed with the DNAnexus platform (DNAnexus, Inc., Mountain View, CA) using default parameters with the human genome assembly hg19 (GRCh37) as reference. Following variant calling, filtering was performed. This included removing variants that was called less that 8 times, were off-target, heterozygous, synonymous, had minor allele frequency (MAF) >1% in ExAC databases (http://exac.broadinstitute.org), and MAF >2% in the Hadassah-Hebrew University Medical Center's internal database (~1200 ethnically matched genomes). Using conservation score and phenotypic relevance to assess the homozygous variants that survived the filtering (**Appendix Table S1**), we discovered Chr5:14690394 G>A; *c.841G>A; p.Gly281Arg* in the *OTULIN* gene. This variant was not present in either the ExAC database (~60,000 genomes: http://exac.broadinstitute.org), the gnomAD database (~125,000 genomes; http://gnomad.broadinstitute.org), or in the Hadassah Hebrew University Medical Center's internal database (~1200 ethnically matched genomes). The mutation was confirmed by Sanger sequencing of two independent PCR products.

Short tandem repeat (STR) analysis of blood cell chimerism

Chimerism was tested by analysis of 16 STR loci from peripheral blood samples from the patient after HSCT. DNA from the patient's post-HSCT blood was amplified using PowerPlex ESI 16 Fast System (Promega, Madison, WI) and compared to samples of isolated donor and patient DNA on a Prism®3130 XL Genetic Analyser (Applied Biosystems, Foster City, CA). The per cent chimerism was calculated from the height and area of the STR amplification peaks according to the manufacturer's instructions.

Multiple sequence alignment

Multiple sequence alignment of OTULIN was performed with the full-length sequences from *Homo sapiens* (human), *Gorilla gorilla (gorilla)* (western lowland gorilla), *Bos taurus* (cow), *Mus musculus* (mouse), *Rattus norvegicus* (rat), *Taeniopygia guttata* (zebra finch), *Gallus gallus* (chicken), *Anolis carolinensis* (green anole lizard), *Xenopus laevis* (African clawed frog), *Danio rerio* (zebrafish), *Petromyzon marinus* (sea lamprey), and *Ciona savignyi* (pacific sea squirt) extracted from the Ensemble database (http://www.ensembl.org/index.html) using Clustal Omega (https://www.ebi.ac.uk/Tools/msa/clustalo). Residue numbering is relative to human OTULIN and secondary structures were annotated according to the structure of human OTULIN in complex with M1-linked diUb (PDB: 3ZNZ) (Keusekotten *et al*, 2013) using ESPript 3.0 (http://espript.ibcp.fr). Blue shading indicates completely conserved residues.

Plasmids and cloning

Constructs encoding the Glutathione-S-transferase (GST)-tagged TUBE (tandem Ub binding entity) have been described previously (Hrdinka *et al*, 2016; Fiil *et al*, 2013).

Constructs for the recombinant expression of OTULINcat and OTULINcat^{C129A} have been described previously (Keusekotten *et al*, 2013). OTULINcat^{G281R} was generated from overhanging primers and confirmed by Sanger sequencing.

Expression and purification of recombinant proteins

Human OTULIN constructs and the TUBE construct were expressed in *E. coli* strain Rosetta2 (DE3) pLacI. Cells were grown at 37 °C in 2 xTY medium containing 30 mg/mL kanamycin or 100 μg/mL Ampicillin (Formedium Ltd., Hunstanton, UK) and 34 mg/mL chloramphenicol (Alfa Aesar, Haverhill, MA) to an OD_{600} of 0.8. The cultures were cooled to 18 °C before induction with 400 μM IPTG (Melford Laboratories Ltd., Ipswich, UK) and harvested 20 h after induction. Cells were resuspended and lysed by sonication in lysis buffer (for OTULIN: 20 mM Tris pH 7.4, 300 nM NaCl, 2 mM β-mercaptoethanol, and 40 mM imidazole; for TUBE: 25 mM Tris pH 8.5, 200 mM NaCl, and 2 mM β-mercaptoethanol; both buffers were supplemented with DNAse I (Sigma, St. Louis, MO), lysozyme (Sigma), and cOmplete protease inhibitor cocktail (Roche, Mannheim, Germany)). OTULIN was purified by immobilised metal affinity chromatography using a HisTrap HP column (GE Healthcare, Milwaukee, WI). The His6 tag was cleaved by overnight incubation with 3C protease, in anion exchange dialysis buffer (20 mM Tris pH 8.0, 4 mM DTT). The protein was further purified by anion exchange chromatography (ResourceQ, GE Healthcare) and the eluted OTULIN was subjected to size exclusion chromatography (HiLoad 16/60 Superdex 75, GE Healthcare) in buffer containing: 20 mM Tris pH 8.0, 150 mM NaCl, 4 mM DTT. TUBE was purified using TALON metal affinity resin (Clontech, Fremont, CA) and eluted in lysis buffer supplemented with 300 mM imidazole. Eluted protein was

further purified by size exclusion chromatography (HiLoad 16/60 Superdex 75, GE Healthcare) in PBS containing 10% glycerol and 1 mM DTT.

Qualitative DUB assay

Qualitative Ub cleavage assays were performed as previously described (Keusekotten *et al*, 2013). Briefly, OTULINcat^{WT} and OTULINcat^{G281R} at several concentrations were diluted in 25 mM Tris pH 7.4, 150 mM NaCl, and 10 mM DTT and incubated with 1 μM tetraUb in DUB buffer (50 mM Tris pH 7.4, 50 mM NaCl, 5 mM DTT) at 37 °C. Samples were taken at different time points and mixed with 4 x SDS sample buffer to stop the reaction. Samples were resolved by SDS-PAGE on 4-12% NuPAGE gels (Life Technologies, Carlsbad, CA) and visualised by silver staining (SilverStain Plus kit, Bio-Rad, Hercules, CA).

Protein crystallization and structure determination

OTULINcat^{G281R} crystals were grown by hanging drop vapour diffusion from drops containing 1 μl each of protein and reservoir (100 mM HEPES, 18% (w/v) PEG 6k, 1M LiCl). Prior to vitrification crystals were transferred into reservoir solution containing 28% (v/v) glycerol, pH 7.4). Diffraction data were collected at Diamond Light source beamline I03. Diffraction images were processed using xia2 (Winter, 2010) and manually scaled using AIMLESS (Evans & Murshudov, 2013). The structure of OTULINcat^{G281R} was determined by molecular replacement using PHASER (McCoy *et al*, 2007) with wild-type OTULINcat (PDB ID 3ZNV) as a search model. Iterative rounds of model building and refinement were performed with COOT (Emsley *et al*, 2010) and PHENIX (Adams *et al*, 2011), respectively. Data collection

and refinement statistics can be found in **Appendix Table S2**. All structure figures were generated with Pymol (www.pymol.org).

Binding assays

To measure binding affinities of OTULINcat^{C129A} and OTULINcat$^{C129A/G281R}$ to M1-diUb, fluorescence anisotropy experiments were performed as previously described (Keusekotten *et al*, 2013). In brief, 10 μL of 100 nM FlAsH-tagged M1-diUb were dispensed in a 384-well Corning flat-bottom plate. Serial dilutions of OTULINcat^{C129A} and OTULINcat$^{C129A/G281R}$ were prepared in FlAsH buffer (20 mM Tris pH 7.4, 200 mM NaCl, 2 mM β-mercaptoethanol, 0.05 % (v/v) Tween-20) and 10 μL were added to FlAsH-tagged M1-diUb containing wells. Fluorescence polarization was recorded on a PheraStar plate reader (BMG Labtech, Aylesbury, UK) using an optics module with λ_{ex} = 485 nm and λ_{em} = 520 nm, and values were fitted to a one-site total binding model using Graphpad Prism 6 to derive binding constants (k_D).

Differential scanning fluoritmetry (DSF) thermal unfolding experiments

Nano-DSF measurements were performed using a Prometheus NT.48 instrument (NanoTemper Technologies GmbH, Munich, Germany). Experiments on OTULINcat^{WT} and OTULINcat^{G281R} were performed at 1.0 mg/mL, 0.4 mg/mL, and 0.1 mg/mL yielding identical results. Only data from measurements at 1.0 mg/ml are shown. Samples were dialysed into buffer containing: 20 mM Na/K phosphate, pH 7.4, 200 mM NaCl, 0.5 mM TCEP before measurement and 10 μL of each sample were loaded in UV capillaries (NanoTemper Technologies). Temperature gradient was set at 2.5 °C/min in a range from 20 to 90 °C. Protein unfolding was measured

by detection of change in tryptophan fluorescence at emission wavelengths of 300 and 350 nm, dependent on temperature gradient. Melting temperatures (T_m) were calculated according to the manufacturer's instructions.

Mouse Embryonic Fibroblasts (MEFs)

Primary MEFs were isolated from E13.5 *Cre*-ERT2-*Otulin*$^{-/flox}$ embryos (Damgaard *et al*, 2016). Briefly, embryos were decapitated, and foetal livers removed, before the embryos were minced and trypsinised using TrypLE Express (Life Technologies) for 30 min at 37 °C. The slurry was passed through a 70 μm cell strainer and plated in tissue culture flasks in high glucose DMEM + GlutaMAX™ (Life Technologies) supplemented with 10% FCS (Life Technologies), and Penicillin/Streptomycin (Life Technologies). After two passages, the MEFs were immortalised using retrovirus carrying SV40 Large T antigen produced in HEK293T Phoenix amphitrophic packaging cells transfected with the pBABE-SV40-Puro and pCL-Eco plasmids (Naviaux *et al*, 1996). Primary MEFs were infected using 50% fresh complete culture medium and 50% retrovirus-containing medium. Immortalised clones were selected for 10 days in complete culture medium with 10 μg/mL puromycin (Invivogen, San Diego, CA). For deletion of OTULIN, the immortalised *Cre*-ERT2-*Otulin*$^{-/flox}$ MEFs were cultured in the presence of 100 nM 4-hydroxytamoxifen (4-OHT) (Sigma) dissolved in 70% ethanol, or with ethanol alone, for one week and cultured for an additional three weeks in complete culture medium alone.

Ub Chain Composition Analysis (AQUA-MS)

TUBE purified Ub chains were separated on a NuPAGE 4-12% gradient gel (Life Technologies) before in-gel digestion with sequencing-grade modified trypsin (Promega) and the addition of 2 picomole of each Ub AQUA peptide internal standard as described previously (Kirkpatrick *et al*, 2006; Shevchenko *et al*, 2006). AQUA peptides were obtained from Cell Signaling Technology, Danvers, MA. Peptides were extracted from gel slices, lyophilized and stored at -80 °C. Peptides were resuspended in 15 μL of reconstitution buffer (1% ACN, 0.5% TFA, 0.01% H2O2). Oxidation of peptides containing methionine residues was performed according to (Phu *et al*, 2011). 10 μL of each sample was loaded onto a nanoEase MZ Symmetry trap (C18, 5 μm, 100 Å, 180 µm x 20 cm; Waters, Milford, MA) at a flow rate of 300 nL/min using a Dionex UltiMate 3000 HPLC system (Thermo Fisher Scientific, Waltham, MA). Peptides were eluted from the trap using a 25 min ACN gradient (2.5-35%). Immediately prior to ionisation, peptides were further separated using an nanoEase MZ HSS T3 column (1.8 μm, 100 Å, 75 µm x 25 cm; Waters). Analysis was performed on a Q-Exactive mass spectrometer (Thermo Fisher Scientific) using parallel reaction monitoring (PRM), as in (Wauer *et al*, 2015). For PRM assays, monoisotopic precursor masses were isolated (2 m/z window) and fragmented at scheduled chromatographic retention times. Precursor masses were fragmented using the following settings: resolution, 17,500; AGC target, 1E5; maximum injection time, 120 ms; normalised collision energy, 28. Raw files were searched and fragment ions quantified using Skyline version 2.5.0.6157© (MacLean *et al*, 2010). Data generated from Skyline was exported into a Microsoft Excel spread sheet for further analysis according to (Kirkpatrick *et al*, 2006).

Purification of endogenous polyUb conjugates by TUBE pulldown

Endogenous polyUb conjugates were purified from primary fibroblasts using TUBE affinity reagents as described previously (Fiil *et al*, 2013). Briefly, cells were lysed in buffer containing 20 mM Na_2HPO_4, 20 mM NaH_2PO_4, 1% (v/v) NP-40, and 2 mM EDTA and supplemented with 5 mM NEM, and cOmplete protease inhibitor cocktail (Roche). TUBE (50 μg/ml) was added directly to the lysis buffer immediately before lysis and cells were scraped off the plates, mixed by pipetting five times, and lysed on ice for 30 min. Lysate was cleared by centrifugation and Glutathione Sepharose 4B beads (GE Healthcare) were added and pulldown performed for 4-16 h at 4 °C on rotation. The beads were then washed four times in 1 mL of ice-cold PBS + 0.1% (v/v) Tween-20 and bound material was eluted by mixing the beads with 1x sample buffer and heating to 50 °C for 5 min.

Immunoprecipitation of the TNF-RSC.

Immunoprecipitation of the TNF receptor signalling complex (TNF-RSC) was performed as previously described (Fiil *et al*, 2013). Briefly, primary fibroblasts were stimulated with 100 ng/mL FLAG-tagged TNF (Enzo Life Sciences, Farmingdale, NY) as indicated, washed in ice-cold PBS, and lysed in 30 mM Tris pH 7.4, 120 mM NaCl, 2 mM KCl, 1% Triton-X-100, 2 mM EDTA, 5 mM *N*-methylmaleimide (NEM), and 1x cOmplete protease inhibitor (Roche) for 30 min on ice. Lysates were cleared by centrifugation and incubated at 4 °C for 16 h with anti-FLAG (M2) agarose beads (Sigma). For time point 0 samples, 100 ng FLAG-TNF was added to the cleared lysates. Beads were washed five times in 1 mL of lysis buffer and bound proteins

were eluted by mixing the beads with 1x sample buffer and heating to 50 °C for 5 min.

MTT reduction assay for cell viability

MTT reduction assay was performed as previously described (Damgaard *et al*, 2013). Briefly, primary fibroblasts or THP-1 cells were seeded in 96-well plates, 24 h before treatment with recombinant human TNF (100 ng/mL; Life Technologies), ultrapure LPS from *E. coli* K12 (100 ng/mL; Invivogen), poly(I:C) (1 μg/mL; Invivogen), Stauroporine (1 μM, Sigma), cycloheximide (CHX; 50 μg/mL; Santa Cruz Biotechnology), or a combination of recombinant human TNF and CHX with or without the caspase inhibitor Q-VD-OPh (10 μM; BioVision, Milpitas, CA) or the RIPK1 kinase inhibitor Necrostatin-1 (Nec-1) (10 μM; Cayman Chemical, Ann Arbor, MI) as indicated. Medium was aspirated and 100 μL fresh medium was added together with 25 μL 3-(4,5-dimethylthiazol-2-yl)-2,5-diphenyltetrazoliym bromide (MTT; Sigma, 5 mg/mL dissolved in PBS). Cells were then left for 1.5-2 h at 37 °C in the dark. Afterwards, 100 μL solubilisation buffer (20% SDS (w/v) dissolved in 50% *N,N*-dimethylformamide) was added and samples were left in the dark over night. Absorbance at 570 nm was read with a reference filter of 620 nm. Individual experiments were performed in duplicate. Data were normalized to untreated samples.

Quantitative Real-Time PCR

Total RNA was extracted from primary skin fibroblasts using the RNeasy Mini Kit (QIAGEN), and DNase digestion was performed on-column with the RNase-Free

DNase Set (QIAGEN) according to the manufacturer's protocol. Total RNA was reverse transcribed using Quantitect Reverse Transcription Kit (QIAGEN). RT-PCR was performed using QuantiFast SYBR Green RT-PCR Kit (QIAGEN) on a Viia7 Real-Time PCR Instrument (Applied Biosystems) with the primers indicated below. Each sample was run in duplicate. Results were normalised to those of 18S rRNA as internal housekeeping control using the 2^(-ΔCt)-method. Primers were: 18S *rRNA* 5'-GTAACCCGTTGAACCCCATT-3' and 5'-CCATCCAATCGGTAGTAGCG-3'; *OTULIN* 5'- AAAGAGGGGCATCAGAACCG-3' and 5'-GGCCCTCAGTGCACAGTAAT-3'; *HOIP* 5'-ACCCCCTATTGAGAGAGATTGCT-3'and 5'-TGGAGCCTGGGACAGAGG-3'; *SHARPIN* 5'-GAAGCCTCCACACTCAAG-3' and 5'-TTCTCTCTCCGTCAAGTTTC-3'; *HOIL-1* 5'-CGATTCAGCAGTATTTAC-3' and 5'-GACTTAGAGAATGAACATC-3'.

Immunoblotting

Samples were resolved on 4-12% Bis-Tris NuPAGE gels (Life Technologies) and transferred to nitrocellulose or polyvinylidene fluoride (PVDF) membranes using the Trans-Blot Turbo transfer system (Bio-Rad). Membranes were blocked in 5% (w/v) skimmed milk in PBS-T (PBS + 0.1% (v/v) Tween-20) for 30 min and incubated with primary antibodies in PBS-T + 3% (w/v) BSA (Sigma) at 4 °C over night. Afterwards, immunoblots were washed in PBS-T and incubated at room temperature for 1 h with HRP-coupled secondary antibodies and then visualised using Clarity™ Western ECL Substrate (Bio-Rad) on a ChemiDoc™ Touch or ChemiDoc™ MP imager (Bio-Rad). Digital images were processed using the Image Lab software (Bio-Rad). Densitometry analysis of immunoblots was performed using the Fiji software

(Schindelin *et al*, 2012). Please see **Appendix Table S3** for details on antibodies used in this study.

chr	chrStart	chrEnd	Ref Seq	Var Seq	varType	cover	rsidList	GERP	genesList	Mutation Taster	Mutation Taster score	ExAC MAF	Gene list component	protImpactList	aaChange	Global Allele Freq	exac het	exac hom	traitList
4	113568496	113568496	T	C	SNP (x2)	28		-2.49	LARP7	N	1	.	CDS	MISSENSE	I263T;I270T	0	0	0	Alazami syndrome
5	14690394	14690394	G	A	SNP (x2)	26		6.07	OTULIN	D	1	.	CDS	MISSENSE	G281R	0	0	0	Autoinflammation, panniculitis, and dermatosis syndrome (AIPDS) / ORAS
4	120057699	120057699	A	G	SNP (x2)	42		-2	MYOZ2	N	0.688	0.000008454	CDS	MISSENSE	M7V	0.01	1	0	Cardiomyopathy, familial hypertrophic, 16
5	148407272	148407272	C	T	SNP (x2)	37		1.27	SH3TC2	N	1	0.0001	CDS	MISSENSE	V222I;V675I; V668I;V560I	0.01	14	0	Charcot-Marie-Tooth disease, type 4C; Mononeuropathy of the median nerve, mild
7	36438952	36438952	T	C	SNP (x2)	53		5.55	ANLN	D	1	.	CDS	MISSENSE	M146T	0	0	0	Focal segmental glomerulosclerosis 8
5	142779875	142779875	T	C	SNP (x2)	126		0.384	NR3C1	N	0.976	0.0000499	CDS;INTRON	MISSENSE	N177S	0.01	6	0	Glucocorticoid resistance
4	126239421	126239421	C	T	SNP (x2)	78	rs202125547	3.03	FAT4	D	1	0.0003	CDS	MISSENSE	R619C	0.03	34	0	Hennekam lymphangiectasia-lymphedema syndrome 2
8	21978269	21978269	C	T	SNP (x2)	25	rs114871775	0.547	HR	N	1	0.0119	CDS	MISSENSE	R857H	0.22	50	0	Hypotrichosis 4; Atrichia with papular lesions; Alopecia universalis congenita
5	138362532	138362532	C	G	SNP (x2)	72		1.53	SIL1	D	0.73	.	CDS	MISSENSE	E201D;E208 D	0	0	0	Marinesco-Sjogren syndrome
16	53690423	53690423	G	T	SNP (x2)	38	rs79524027	5.82	RPGRIP1L	D	1	0.0003	CDS	MISSENSE	L554I	0.03	35	0	Meckel syndrome 5; Joubert syndrome 7; COACH syndrome; Retinal degeneration in ciliopathy
5	14508274	14508274	G	A	SNP (x2)	135		5.73	TRIO	N	1	.	CDS	MISSENSE	D3013N;D28 37N;D512N	0	0	0	Mental retardation, autosomal dominant 44
4	104103975	104103975	G	A	SNP (x2)	40	rs115429647	4.68	CENPE	D	1	0.000008247	CDS	MISSENSE	R-303-C	0.01	1	0	Microcephaly 13, primary, autosomal recessive
2	145157395	145157395	C	T	SNP (x2)	77		4.46	ZEB2	N	0.867	.	CDS	MISSENSE	M453I;M429I	0	0	0	Mowat-Wilson syndrome
8	23560406	23560406	C	A	SNP (x2)	42	rs534202593	3.47	NKX2-6	D	0.947	0.00009398	CDS	MISSENSE	R73L;R155L	0.01	2	0	Persistent truncus arteriosus; Conotruncal heart malformations
8	23560511	23560511	C	G	SNP (x2)	14	rs61743032	3.58	NKX2-6	N	0.992	0.0019	CDS	MISSENSE	R38P;R120P	0.13	22	0	Persistent truncus arteriosus; Conotruncal heart malformations
1	11298552	11298552	T	C	SNP (x2)	135	rs138066958	4.35	MTOR	D	1	0.0003	CDS	MISSENSE	I637V	0.03	34	0	Smith-Kingsmore syndrome

Enlis Genome Research version 1.9

Table S1. Genetic variants identified by whole exome sequencing.

	OTULIN 80-352 G281R
**Data collection**	
Beamline	Diamond I03
Space group	P 2_1 2_1 2_1
a, b, c, (Å)	43.26, 72.44, 93.73
α, β, γ (°)	90, 90, 90
Wavelength (Å)	0.97624
Resolution (Å)	72.44 - 1.77 (1.81 - 1.77)
R_{merge}	5.4 (77.4)
< I / σI >	16.3 (2.3)
CC (1/2)	1.00 (0.74)
Completeness (%)	98.6 (100.0)
Redundancy	6.5 (6.6)
**Refinement**	
Resolution (Å)	57.32 - 1.77
No. reflections	29,002
R_{work} / R_{free}	16.2 / 17.9
No. atoms	
Protein	4,333
Ligand / ion	53
Water	253
B-factors	
Wilson B	28.06
Protein	34.82
Ligand / ion	63.08
Water	43.57
R.m.s. deviations	
Bond length (Å)	0.006
Angels (°)	0.774
Ramachandran statistics (outliers, allowed, favoured)	0.0, 3.1, 96.9

Appendix Table S2. Data collection and refinement statistics. Numbers in brackets are for the highest resolution bin.

Antibody	Catalog #	Clone	RRDI	Supplier	Dilution
OTULIN	14127		AB_2576213	Cell Signaling Technology	1:1000
OTULIN	ab151117		AB_2728115	Abcam	1:1000
IκBα	9242		AB_10694550	Cell Signaling Technology	1:1000
phospho-IκBα (S32)	2859		AB_561111	Cell Signaling Technology	1:500
p65/RelA	8242		AB_10859369	Cell Signaling Technology	1:2500
phospho-p65/RelA (S563)	3033		AB_331285	Cell Signaling Technology	1:2500
NEMO/IKKγ	sc-8330		AB_2124846	Santa Cruz Biotechnology	1:1000
HOIP/RNF31	ab46322		AB_945269	Abcam	1:500
HOIL-1/RBCK1	MABC576	2E2	AB_2737058	Merck Millipore	1:1000
HOIL-1/RBCK1	NBP1-88301		AB_11018853	Novus Biologicals	1:1000
SHARPIN	14626-1-AP		AB_2187734	ProteinTech	1:2000
TNF-R1	sc-8436	H-5	AB_628377	Santa Cruz Biotechnology	1:500
TNF-R1	ab19139		AB_2204128	Abcam	1:500
CYLD	sc-74435		AB_1122022	Santa Cruz Biotechnology	1:200
CYLD	8462		AB_10949157	Cell Signaling Technology	1:1000
p38	9212		AB_330713	Cell Signaling Technology	1:1000
phospho-p38 (T180/Y182)	9216		AB_331296	Cell Signaling Technology	1:500
JNK	9258		AB_2141027	Cell Signaling Technology	1:1000
phospho-JNK (T183/Y185)	4668		AB_823588	Cell Signaling Technology	1:500
Ubiquitin	NB300-130	Ubi-1	AB_2238516	Novus Biologicals	1:1000
M1-linked/linear ubiquitin	MABS199	1E3	AB_2576212	Merck Millipore	1:500
M1-linked/linear ubiquitin	AB130	LUB9	AB_2576211	LifeSensors	1:500
Actin	MAB1501R	C4	AB_2223041	Merck Millipore	1:10,000
anti-rabbit IgG-HRP	NA934		AB_772206	GE Healthcare	1:5000
anti-mouse IgG-HRP	NXA931		AB_772209	GE Healthcare	1:5000
anti-mouse IgG light chain-HRP	AP200P		AB_805324	Merck Millipore	1:2000

Appendix Table S3. Primary and secondary antibodies. The target, catalog number (#), clone, Research Resource Identifier (RRID), supplier, and dilution used for primary and secondary antibodies used in this study.

Supplementary References

Adams PD, Afonine PV, Bunkóczi G, Chen VB, Echols N, Headd JJ, Hung L-W, Jain S, Kapral GJ, Grosse-Kunstleve RW, McCoy AJ, Moriarty NW, Oeffner RD, Read RJ, Richardson DC, Richardson JS, Terwilliger TC & Zwart PH (2011) The Phenix software for automated determination of macromolecular structures. *Methods* **55:** 94–106

Damgaard RB, Fiil B, Speckmann C, Yabal M, Stadt zur U, Bekker-Jensen S, Jost PJ, Ehl S, Mailand N & Gyrd-Hansen M (2013) Disease-causing mutations in the XIAP BIR2 domain impair NOD2-dependent immune signalling. *EMBO Mol Med* **5:** 1278–1295

Damgaard RB, Walker JA, Marco-Casanova P, Morgan NV, Titheradge HL, Elliott PR, McHale D, Maher ER, McKenzie ANJ & Komander D (2016) The Deubiquitinase OTULIN Is an Essential Negative Regulator of Inflammation and Autoimmunity. *Cell* **166:** 1215–1230.e20

Emsley P, Lohkamp B, Scott WG & Cowtan K (2010) Features and development of Coot. *Acta Crystallogr. D Biol. Crystallogr.* **66:** 486–501

Evans PR & Murshudov GN (2013) How good are my data and what is the resolution? *Acta Crystallogr. D Biol. Crystallogr.* **69:** 1204–1214

Fiil B, Damgaard RB, Wagner SA, Keusekotten K, Fritsch M, Bekker-Jensen S, Mailand N, Choudhary C, Komander D & Gyrd-Hansen M (2013) OTULIN Restricts Met1-Linked Ubiquitination to Control Innate Immune Signaling. *Mol Cell* **50:** 818–830

Hrdinka M, Fiil B, Zucca M, Leske D, Bagola K, Yabal M, Elliott PR, Damgaard RB, Komander D, Jost PJ & Gyrd-Hansen M (2016) CYLD Limits Lys63- and Met1-Linked Ubiquitin at Receptor Complexes to Regulate Innate Immune Signaling. *Cell Rep* **14:** 2846–2858

Keusekotten K, Elliott PR, Glockner L, Fiil B, Damgaard RB, Kulathu Y, Wauer T, Hospenthal MK, Gyrd-Hansen M, Krappmann D, Hofmann K & Komander D (2013) OTULIN Antagonizes LUBAC Signaling by Specifically Hydrolyzing Met1-Linked Polyubiquitin. *Cell* **153:** 1312–1326

Kirkpatrick DS, Hathaway NA, Hanna J, Elsasser S, Rush J, Finley D, King RW & Gygi SP (2006) Quantitative analysis of in vitro ubiquitinated cyclin B1 reveals complex chain topology. *Nat Cell Biol* **8:** 700–710

MacLean B, Tomazela DM, Shulman N, Chambers M, Finney GL, Frewen B, Kern R, Tabb DL, Liebler DC & MacCoss MJ (2010) Skyline: an open source document editor for creating and analyzing targeted proteomics experiments. *Bioinformatics* **26:** 966–968

McCoy AJ, Grosse-Kunstleve RW, Adams PD, Winn MD, Storoni LC & Read RJ

(2007) Phaser crystallographic software. *J Appl Crystallogr* **40:** 658–674

Naviaux RK, Costanzi E, Haas M & Verma IM (1996) The pCL vector system: rapid production of helper-free, high-titer, recombinant retroviruses. *J Virol* **70:** 5701–5705

Phu L, Izrael-Tomasevic A, Matsumoto ML, Bustos D, Dynek JN, Fedorova AV, Bakalarski CE, Arnott D, Deshayes K, Dixit VM, Kelley RF, Vucic D & Kirkpatrick DS (2011) Improved quantitative mass spectrometry methods for characterizing complex ubiquitin signals. *Molecular & cellular proteomics : MCP* **10:** M110.003756

Shevchenko A, Tomas H, Havlis J, Olsen JV & Mann M (2006) In-gel digestion for mass spectrometric characterization of proteins and proteomes. *Nature protocols* **1:** 2856–2860

Wauer T, Simicek M, Schubert A & Komander D (2015) Mechanism of phospho-ubiquitin-induced PARKIN activation. *Nature* **524:** 370–374

Winter G (2010) xia2: an expert system for macromolecular crystallography data reduction. *J Appl Crystallogr* **43:** 186–190
