# Supplementary material for: OTULIN deficiency in ORAS causes cell type‐specific LUBAC degradation, dysregulated TNF signalling and cell death
Source: EMBO Mol Med. 2019 Feb 25;11(3):e9324. doi: 10.15252/emmm.201809324 (PMC6404114; doi:10.15252/emmm.201809324)
Supplement: Supplementary file 2 — Expanded View Figures PDF [file EMMM-11-e9324-s002.pdf]

Expanded View Figures

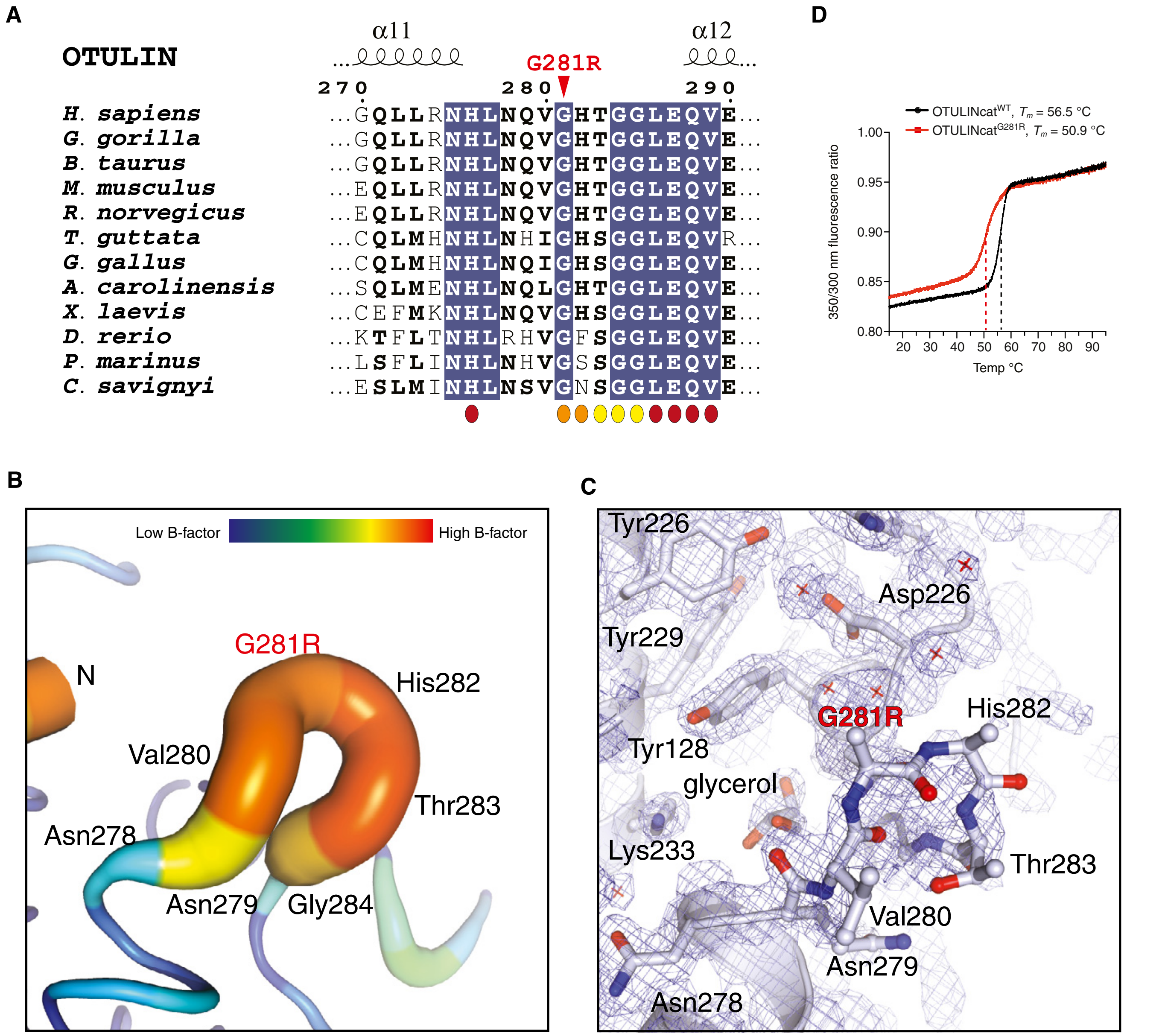

Figure EV1. Structural analysis of OTULIN^{G281R} (related to Fig 2).

A Multiple sequence alignment of the sequence surrounding Gly281 in OTULIN's catalytic domain. The Gly281Arg mutation is indicated by a red arrowhead. Residues interacting with the proximal (orange), distal (maroon) or both Ub (yellow) moieties are indicated.

B Putty representation of the α11-α12 loop incorporating the Gly281Arg mutation from the OTULIN^{G281R} structure (PDB: 6I9C). An increased radius of the cartoon and increase in colours yellow-red represent increased crystallographic temperature, B factors, and reflect the increased disorder of the α11-α12 loop relative to the rest of the structure (comparable B factors are only found at the N and C termini where there is no secondary structure).

C Electron density for the same region as in (B). A weighted 2Fo-Fc map is shown contoured at 1σ. Residues from the α11-α12 loop are shown as stick representation. Owing to the increased motility of the α11-α12 loop, no interpretable electron density was observed for the side chains of Gly218Arg and His282 and only the Cα and backbone amide could be confidently fitted into the electron density map. Other residues in the region had interpretable electron density and several water molecules in the region were sufficiently ordered to be modelled (red cross).

D Tryptophan fluorescence upon thermal unfolding of OTULINcat^{WT} and OTULINcat^{G281R} (1.0 mg/mL) measured by nanodifferential scanning fluorimetry (nano-DSF). Apparent melting temperatures (T_m) are indicated (dashed lines). Data are representative to two independent experiments.

© 2019 MRC Laboratory of Molecular Biology

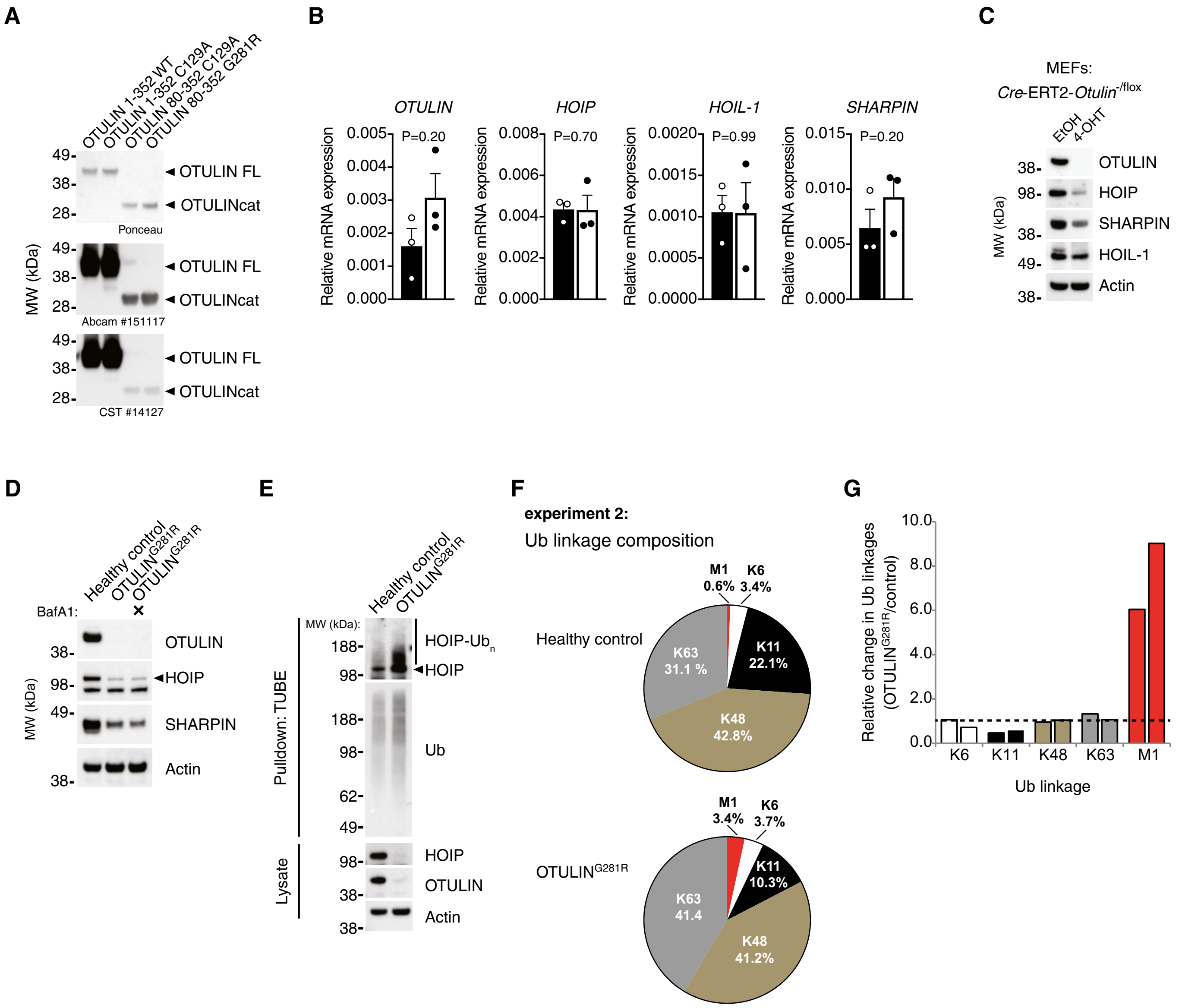

Figure EV2. Analysis of protein and mRNA expression in OTULIN^{G281R} and quantitative proteomics analysis of the cellular Ub linkage composition (related to Fig 3).

A Analysis of antibody recognition of recombinant OTULIN proteins. 200 ng recombinant protein was loaded in each lane. Membranes were stained with Ponceau S before immunoblot analysis was performed with the two anti-OTULIN antibodies used in this study (#14127 from Cell Signaling Technology and ab151117 from Abcam). Data are representative of two independent experiments.

B Relative mRNA levels of *OTULIN, HOIP, HOIL-1 and SHARPIN* from primary healthy control and patient fibroblasts measured by quantitative RT–PCR. Bars represent mean ± SEM of three independent experiments each performed in duplicate. Statistical significance was determined using the Mann–Whitney *U*-test.

C Immunoblot analysis of whole-cell lysates from Cre-ERT2-*Otulin*$^{-/flox}$ inducible knock-out MEFs treated with 4-hydroxytamoxifen (4-OHT) (100 nM) or ethanol (EtOH) alone as indicated. Data are representative of two independent experiments.

D Immunoblot analysis whole-cell lysates from primary healthy control and patient fibroblasts either left untreated or treated with the autophagosome inhibitor bafilomycin A1 (BafA) (100 nM) for 24 h. Data are representative of two independent experiments.

E Immunoblot analysis of endogenous Ub conjugates purified by TUBE pull-down from untreated healthy control and patient fibroblasts shows HOIP ubiquitination in the patient cells. Data are representative of two independent experiments.

F Biological replicate of the experiment shown in Fig 3D. AQUA-MS/MS data from TUBE-based purification of cellular polyUb conjugates from untreated primary fibroblasts from a healthy control or patient III.2 harbouring the OTULIN^{G281R} mutation. K27, K29, linkages could not be detected and K33 could not be accurately quantified in all samples.

G Relative change (OTULIN^{G281R}/control) in the amount of cellular Ub linkages. Bars represent the data presented in Figs 3D and EV2F individually.

Source data are available online for this figure.

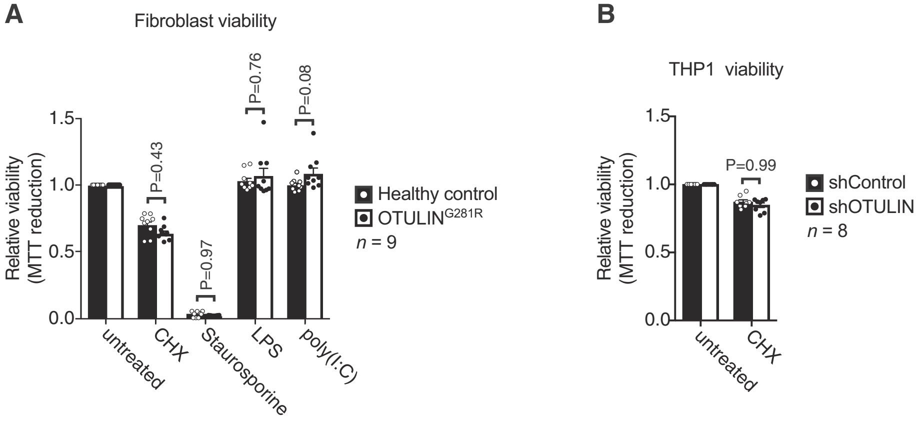

Figure EV3. Analysis of viability of healthy control or OTULIN^{G281R} primary fibroblasts and shControl or shOTULIN THP-1 cells (related to Fig 6).

A Viability of healthy control or OTULIN^{G281R} primary fibroblasts after 24 h of treatment with CHX (50 μg/ml), staurosporine (1 μM), LPS (100 ng/ml) or poly(I:C) (1 μg/ml) as indicated was analysed using MTT reduction assays. Results were normalised to untreated samples. Each experiment was performed in duplicate. Bars represent mean ± SEM (*n* = 9) and were analysed using the two-way ANOVA test of statistical significance with Sidak's correction for multiple comparisons.

B Viability of healthy control or shControl and shOTULIN THP-1 cells after 6 h of combination treatment with TNF (100 ng/ml) and CHX (50 μg/mL) as indicated was analysed using MTT reduction assays. Results were normalised to untreated samples. Each experiment was performed in duplicate. Bars represent mean ± SEM (*n* = 8) and were analysed using the two-way ANOVA test of statistical significance with Sidak's correction for multiple comparisons.

© 2019 MRC Laboratory of Molecular Biology
