# Supplementary figures and images for: OTULIN deficiency in ORAS causes cell type‐specific LUBAC degradation, dysregulated TNF signalling and cell death
Source: EMBO Mol Med. 2019 Feb 25;11(3):e9324. doi: 10.15252/emmm.201809324 (PMC6404114; doi:10.15252/emmm.201809324)

Figure EV2

A

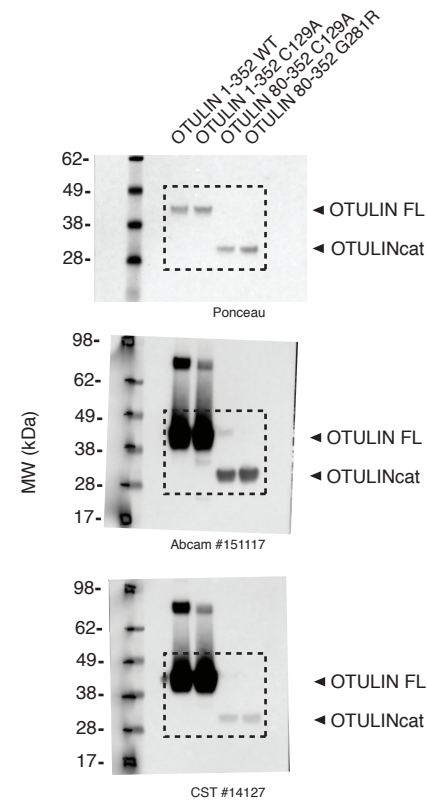

C

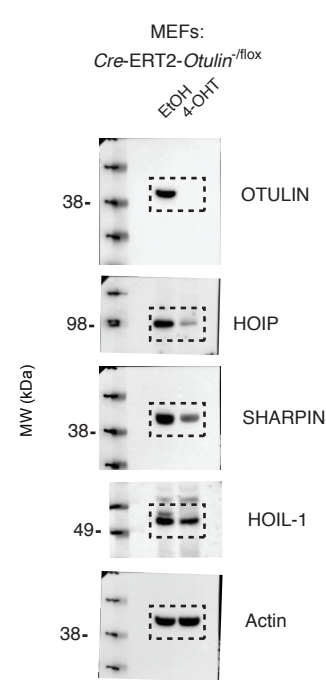

D

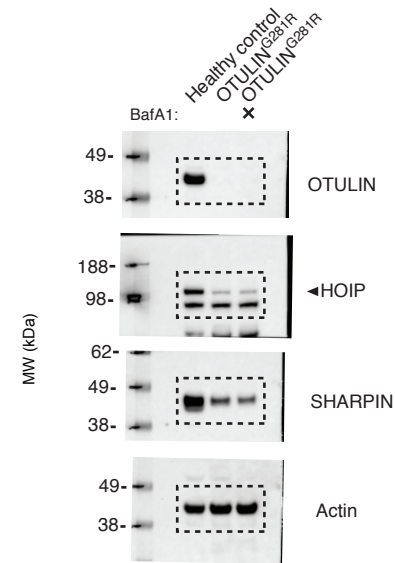

E

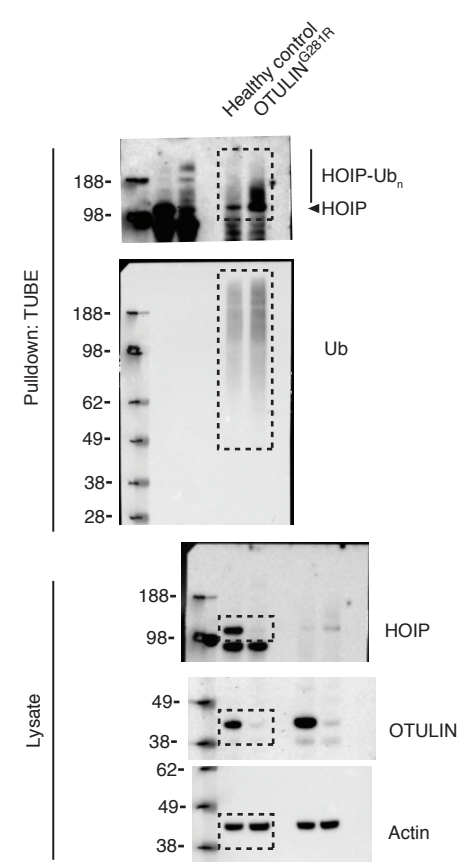

Supplement: Supplementary file 3 — Source Data for Expanded View [file EMMM-11-e9324-s006.zip › emmm201809324-sup-0006-SDataEV/emmm201809324-sup-0006-SDataFigEV2.pdf]

**Figure 3**

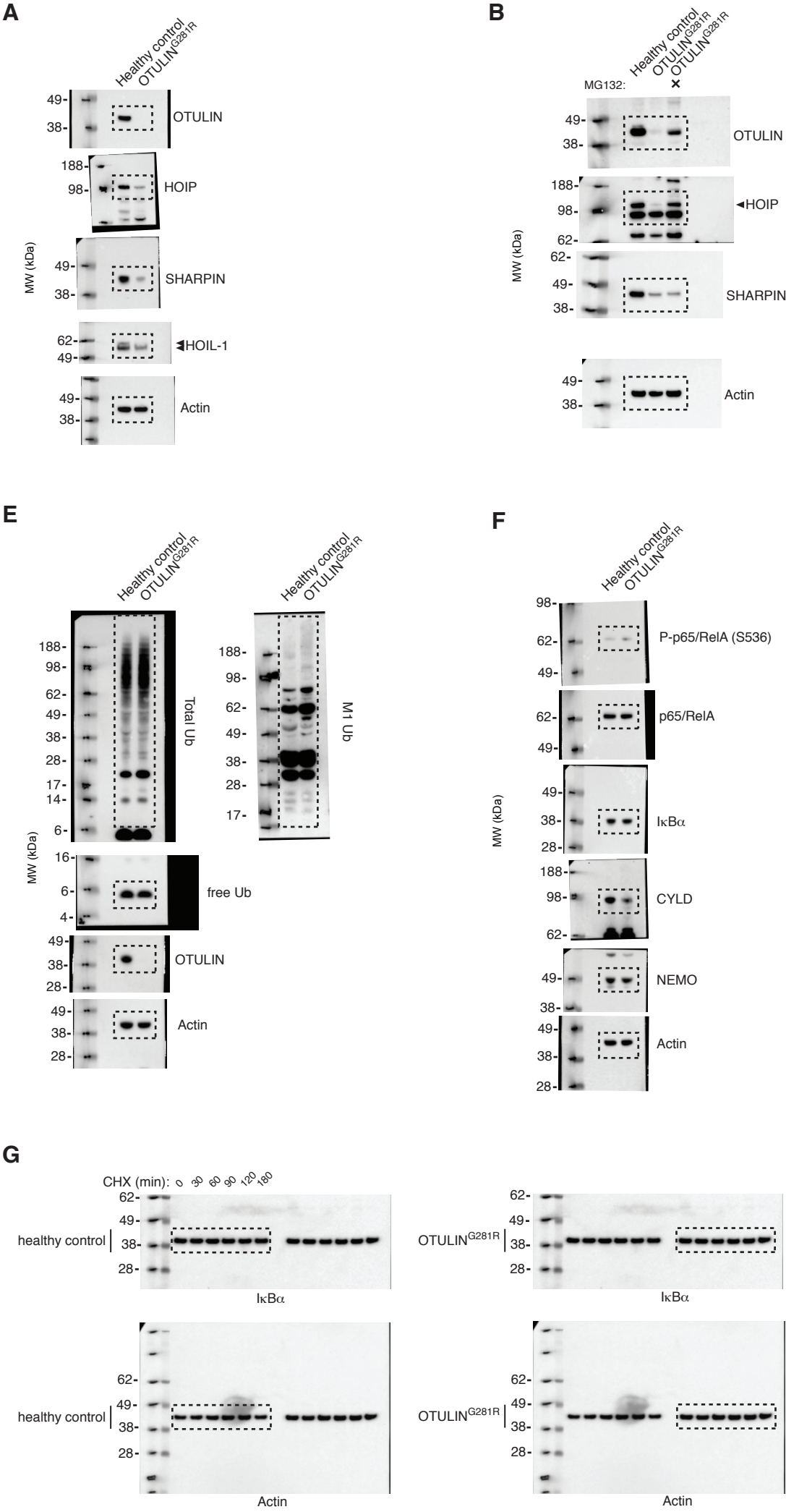

Supplement: Supplementary file 5 — Source Data for Figure 3 [file EMMM-11-e9324-s003.pdf]

Figure 4

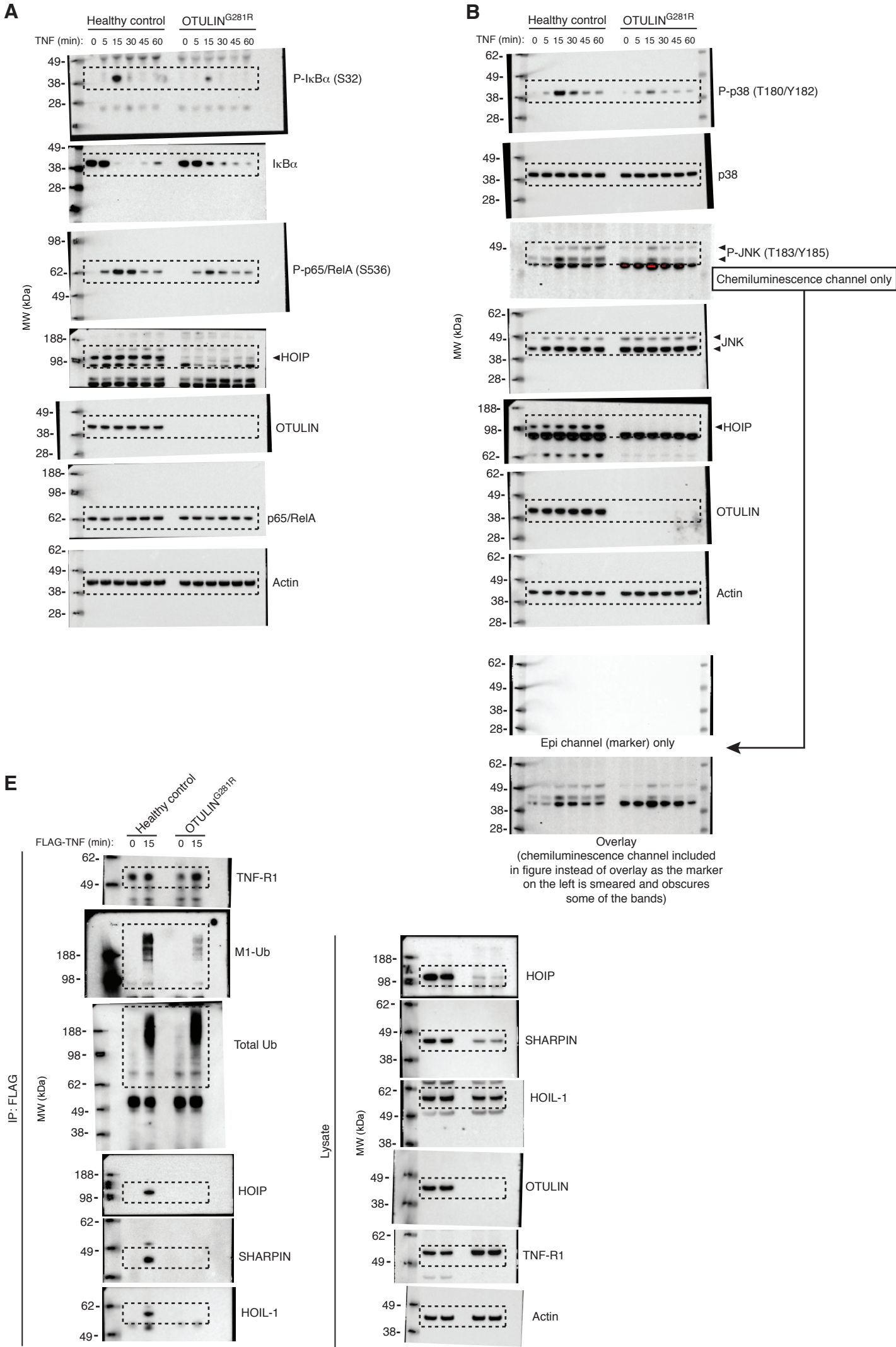

Supplement: Supplementary file 6 — Source Data for Figure 4 [file EMMM-11-e9324-s004.pdf]

Figure 5

A

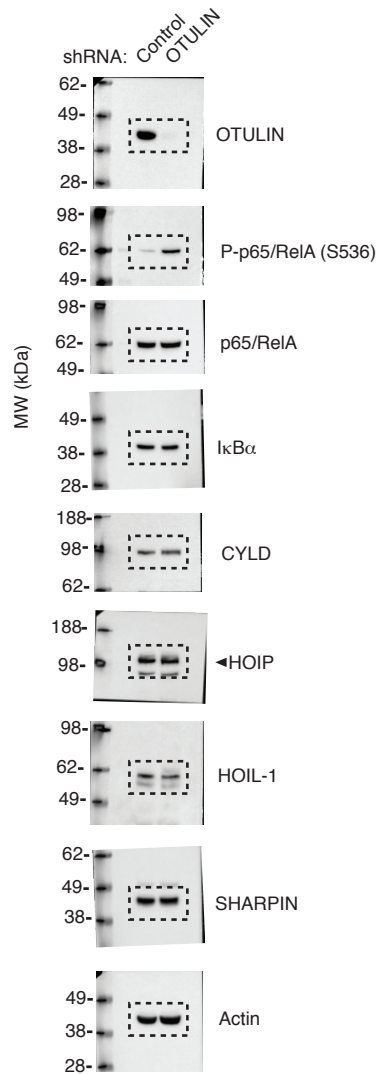

B

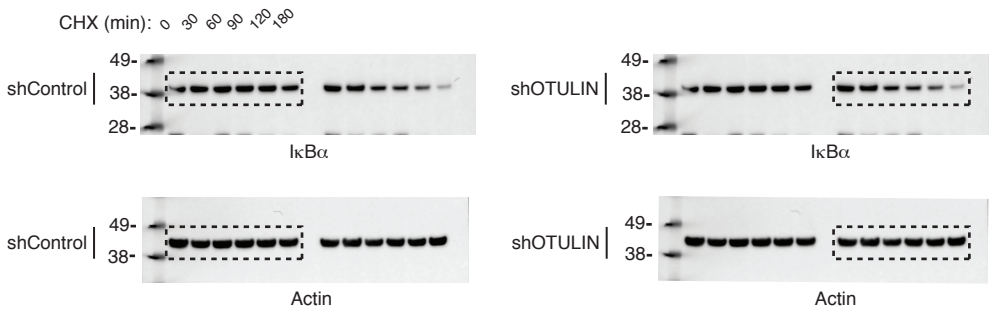

D

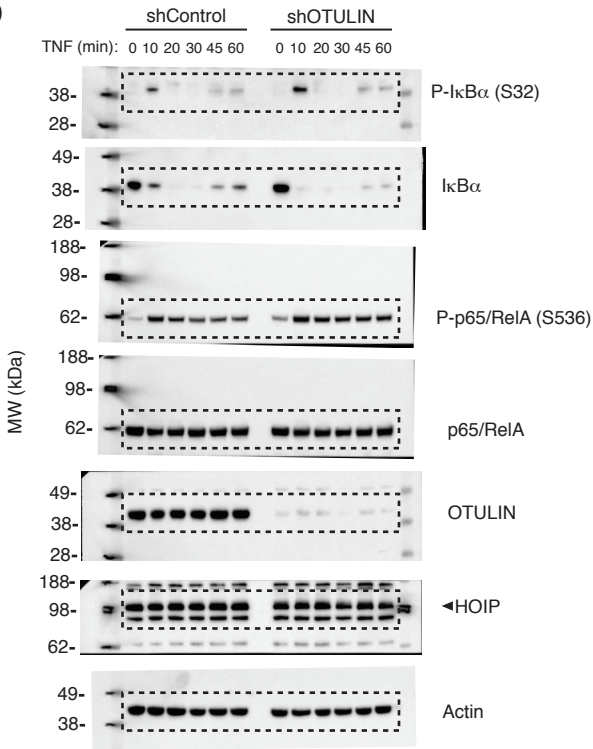

Supplement: Supplementary file 7 — Source Data for Figure 5 [file EMMM-11-e9324-s005.pdf]
